# The impact of kit, environment, and sampling contamination on the observed microbiome of bovine milk

**DOI:** 10.1128/msystems.01158-23

**Published:** 2024-05-24

**Authors:** C. J. Dean, Y. Deng, T. C. Wehri, F. Pena-Mosca, T. Ray, B. A. Crooker, S. M. Godden, L. S. Caixeta, N. R. Noyes

**Affiliations:** 1 Department of Veterinary Population Medicine, University of Minnesota, St. Paul, Minnesota, USA; 2 Department of Animal Science, University of Minnesota, St. Paul, Minnesota, USA; Teagasc Food Research Centre Moorepark, Fermoy, Cork, Ireland

**Keywords:** bovine milk, decontamination, microbiome, mammary gland epithelium, microbial diversity, bacterial culture

## Abstract

In low-microbial biomass samples such as bovine milk, contaminants can outnumber endogenous bacteria. Because of this, milk microbiome research suffers from a critical knowledge gap, namely, does non-mastitis bovine milk contain a native microbiome? In this study, we sampled external and internal mammary epithelia and stripped and cisternal milk and used numerous negative controls, including air and sampling controls and extraction and library preparation blanks, to identify the potential sources of contamination. Two algorithms were used to mathematically remove contaminants and track the potential movement of microbes among samples. Results suggest that the majority (i.e., >75%) of sequence data generated from bovine milk and mammary epithelium samples represents contaminating DNA. Contaminants in milk samples were primarily sourced from DNA extraction kits and the internal and external skin of the teat, while teat canal and apex samples were mainly contaminated during the sampling process. After decontamination, the milk microbiome displayed a more dispersed, less diverse, and compositionally distinct bacterial profile compared with epithelial samples. Similar microbial compositions were observed between cisternal and stripped milk samples, as well as between teat apex and canal samples. *Staphylococcus* and *Acinetobacter* were the predominant genera detected in milk sample sequences, and bacterial culture showed growth of *Staphylococcus* and *Corynebacterium* spp. in 50% (7/14) of stripped milk samples and growth of *Staphylococcus* spp. in 7% (1/14) of cisternal milk samples. Our study suggests that microbiome data generated from milk samples obtained from clinically healthy bovine udders may be heavily biased by contaminants that enter the sample during sample collection and processing workflows.

**IMPORTANCE:**

Obtaining a non-contaminated sample of bovine milk is challenging due to the nature of the sampling environment and the route by which milk is typically extracted from the mammary gland. Furthermore, the very low bacterial biomass of bovine milk exacerbates the impacts of contaminant sequences in downstream analyses, which can lead to severe biases. Our finding showed that bovine milk contains very low bacterial biomass and each contamination event (including sampling procedure and DNA extraction process) introduces bacteria and/or DNA fragments that easily outnumber the native bacterial cells. This finding has important implications for our ability to draw robust conclusions from milk microbiome data, especially if the data have not been subjected to rigorous decontamination procedures. Based on these findings, we strongly urge researchers to include numerous negative controls into their sampling and sample processing workflows and to utilize several complementary methods for identifying potential contaminants within the resulting sequence data. These measures will improve the accuracy, reliability, reproducibility, and interpretability of milk microbiome data and research.

## INTRODUCTION

Animals are hosts to a variety of microbial communities, many of which are site specific, and their structure and function are often unique to a specific anatomical location within the host (1). These site-specific microbiomes can play important roles in the host’s physiology and health through host-microbe and microbe-microbe interactions (2, 3). In dairy cows, the mammary gland is a critical organ due to its central role in milk production (4, 5). The secretory portion of the bovine udder is anatomically complex and composed of four separate mammary glands that contain secretory epithelial cells (i.e., lactogenic cells) capable of synthesizing, storing, and secreting milk components into individual alveoli from which the milk travels through ducts to the gland cistern. Milk produced from a healthy bovine mammary gland was historically believed to be sterile, particularly at its origin high up within individual mammary alveoli (6, 7). However, even in healthy mammary glands, results from culture-independent molecular approaches have cast doubt on the sterility of *in situ* milk because these results show a diverse microbial community profile obtained from 16S rRNA-based workflows (6, 8, 9). The 16S rRNA approach uses sequence variability in certain regions of the 16S rRNA gene to taxonomically classify DNA fragments extracted from a raw sample such as bovine milk (10). Using this approach, previous studies have reported associations between the bovine milk microbiome and immune responses (11), development of mastitis (4), quality and safety of dairy products (12), and the gut microbiota of both calves and consumers (5, 13). Taken together, this body of literature suggests that the milk microbiome is a crucial component of cow health and production as well as milk safety and quality.

Milk is known to be a low-microbial biomass sample matrix, dominated by non-cellular components such as proteins and fat (14). Additionally, the cellular components of milk tend to be dominated by host (i.e., *Bos taurus*) cells as opposed to microbial cells (15, 16). Low-biomass samples are especially challenging for microbiome studies because cells and/or DNA introduced during sample collection and processing can easily outnumber endogenous cells and/or DNA. The challenges of low-biomass sample matrices for microbiome studies have been well documented (17–21). However, even among low-biomass sample matrices, milk has long been noteworthy for the difficulty of obtaining a “clean” sample. One study on the bacterial culture of bovine milk suggested that contamination rates are >10% for quarter milk samples (22). The reason for this high rate of bacterial contamination is the process by which milk is typically sampled, namely, by collecting the so-called “stripped milk.” In this sampling procedure, the external skin of a teat is first thoroughly cleaned and disinfected. Then, the sampler manually expresses and discards several streams of milk from the teat to remove the accumulated somatic cells in the distal teat cistern according to the National Mastitis Council (23). It is thought that this initial milk stream is more likely to contain contaminating bacteria due to the milk flowing through the lower compartments of the gland, i.e., the teat canal, which is more easily accessed by bacteria that can move in a retrograde fashion from the teat apex up into the canal and, if not thwarted by the immune system, from there into the udder cistern, ducts, and mammary tissue. Thus, after discarding the initial milk streams, “aseptic” milk is then collected via continued manual expression from the teat (23). However, even this process cannot eliminate potential contamination of the stripped milk from bacteria and/or DNA located on the external teat epithelium and/or the epithelium that lines the internal teat canal (24, 25). Teat wall puncture is often recommended as the most reliable method of collecting a clean, uncontaminated milk sample (26).

Additionally, even milk not contaminated with bacteria associated with external epithelia can still be contaminated at any of the numerous other downstream harvest and processing procedures, including from exposure to ambient air in the room where samples are being collected. This is a particular concern for most bovine milk microbiome studies, which typically collect samples in large animal handling facilities such as milking parlors or research barns. These environments often contain a relatively high bacterial diversity, which can then contaminate the milk as it is being collected from the teat (4). Furthermore, even a truly aseptically collected sample can be contaminated by DNA contained in molecular extraction and library preparation kits, i.e., the so-called “kitome” (27–29).

Given the numerous pathways by which exogenous bacteria and/or DNA can contaminate a sample, it is expected that most microbiome data sets will contain some level of non-native DNA sequences. Because of this, many microbiome researchers have developed methods and strategies for differentiating exogenous or “contaminating” microbial signatures from the true microbial composition of a sample. Such advancements include additions to study design, inclusion of extensive positive and negative control samples (30), and use of synthetic DNA to allow for absolute quantification of microbes (31). However, it is exceedingly difficult to differentiate contaminating versus endogenous sequences because many bacterial taxa are ubiquitous and could indeed be from both contaminants and endogenous sources within the same sample data set. To address this issue, researchers have leveraged statistical distributions to identify sequences that likely originated from contaminating cells or DNA (32). However, many of these advancements have not yet been fully leveraged for milk microbiome research (28, 33). This gap is particularly important because previous studies have reported that a large proportion of 16S rRNA sequences is “shared” between various udder samples and stripped milk samples (24, 33), highlighting the necessity of accounting for components within the various niches of the udder to understand their contributions to the stripped milk microbiome. Additionally, understanding the origin of sequences in a microbiome data set is critical for interpretation, for example, to understand potential mechanisms that underlie observed epidemiological associations between microbiome dynamics and animal health. If there is a native raw milk microbiome, it is important to study the role it plays in mastitis etiology, in host immune response to pathogens, and in milk quality and safety. Furthermore, it is nearly impossible to develop therapeutic or preventive cow and herd health strategies if the origin of microbes is ambiguous, i.e., whether they originated from the milk itself, from the external or internal epithelia, from the ambient air in the milking parlor, or from lab-based sample handling procedures. To advance dairy microbiome research, we must address the current ambiguity in the literature regarding the “true” versus “contaminating” milk microbiome.

Therefore, the aim of this study was to obtain an accurate description of the true microbial status of the different compartments of the udder and mammary gland in dairy cows without mastitis. To achieve this aim, we used aseptic sampling techniques and positive as well as negative controls during sample collection and processing. Negative controls were used to identify likely bacterial contaminants and their sources using two available statistical algorithms. In addition, propidium monoazide was used to differentiate DNA extracted from intact versus non-intact cells, as DNA from viable cells may have different implications for animal health and milk quality and safety as compared with DNA from non-viable cells (16). We hypothesized that (i) the bovine mammary gland contains a “microbiome gradient” that imparts an increasingly diverse milk microbiome as the milk moves from mammary alveoli through ducts into the gland cistern and internal teat canal and that (ii) current milk sampling techniques artificially inflate the microbial diversity of stripped milk by introducing microbes from the external teat epithelium and ambient air.

## MATERIALS AND METHODS

### Study design and animals

**Fig 1:**
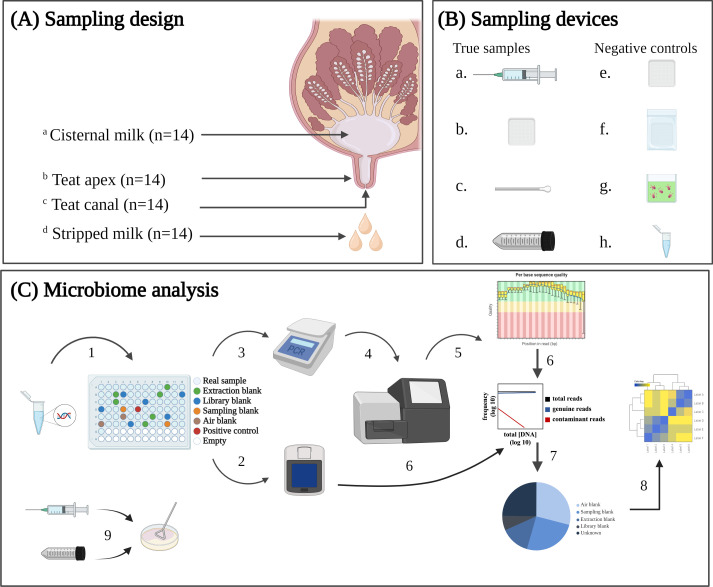
Sampling design (**A**), sample collection devices (**B**), and workflow for microbiome analysis (**C**). Within panels A and B, lowercase letters link sample type with sampling device, (**a**) collection needle, (**b**) gauze square, (**c**) cytobrush swab, (**d**) falcon tube, (**e**) air blank of opened gauze square, (**f**) sampling blank of sealed gauze square, (**g**) extraction blank of lysis buffer, and (**h**) library blank of water. Within panel C, (1) DNA extraction, (2) DNA concentration measurement, (3) quantitative PCR (qPCR) for quantification of the 16S gene, (4) short-read sequencing of the 16S V4 hypervariable region, (5) quality control of sequence data, (6) identification of contaminant sequences, (7) identification of sequence source samples, (8) microbiome analysis, and (9) bacterial culture of cisternal and stripped milk samples. Created with BioRender.com.

This cross-sectional study utilized dairy cows from the University of Minnesota Dairy Cattle Teaching and Research Center. Cows were housed in a tie-stall barn with sawdust-bedded rubber mattresses. Cows were fed non-limiting amounts of a total mixed ration formulated to meet or exceed the nutrient requirements for lactating Holstein cows (34) and consisting of approximately 55% forage and 45% concentrate. Cows were milked in a double 5 herringbone parlor twice daily. History of clinical mastitis was gathered from farm records, in which clinical cases of mastitis diagnosed by farm personnel based on milk appearance were recorded. Somatic cell count (SCC) information was also used to determine eligibility of cows, as we intended to use only cows without clinical or subclinical mastitis. In total, 90 Holstein dairy cows from the herd were assessed for study enrollment on 6 January 2021. Only cows that met the following eligibility criteria were enrolled; in their first, second, or third lactation, >90 days in milk at the time of enrollment; a SCC <200 × 1,000 cells/mL of composite milk for monthly analysis by DHIA, no leaking milk prior to sampling, and no history of clinical mastitis in the last 30 days. Twenty-three cows met these requirements from which 14 cows were randomly selected for enrollment into the study. These 14 cows on average were 182 days in milk (DIM, range from 92 to 456 DIM) and had a milk yield of 38 kg/d (range from 15 to 50 kg/d) and a milk SCC of 91 × 1,000 cells/mL (range from 17 to 325 × 1,000 cells/mL) on the sampling day (Table S1). On the day of sampling, cows in this study were released as a group and milked after all the non-study cows were milked to help ensure that research personnel could aseptically collect milk samples with the minimal amount of potential contamination. From each cow, teat apex, teat canal, stripped milk, and gland cistern milk samples were collected for microbiome analysis (Fig. 1A). In addition, air and sampling blanks were collected in the milking parlor to identify potential microbial contaminants in the downstream sequence data analysis (Fig. 1C). All the procedures and activities of this study were approved by the University of Minnesota Institutional Animal Care and Use Committee (protocol number 1904-36973A).

### Sample collection procedure

All sampling occurred on the same day. A single hindquarter from each cow was randomly selected for sampling to avoid the potential bias between front and hind quarters. Disposable gloves were used and discarded between each sample type and between each sampled cow. Manure was removed from the tie-stalls at least twice daily, and fresh bedding was added while the cows were being milked. This stall management helped keep udders primarily dry and clear of substantial debris, so the selected hindquarters required no cleaning prior to sampling. Teat apex and teat canal samples were collected before the milk samples to avoid potential contamination from residual milk that would coat the epithelium of the streak canal after collection of the milk. Teat apex samples were collected first from the distal one-third of the apex, using a sterile, pre-moistened gauze square soaked in germ-free phosphate buffer saline solution (PBS) (Fig. 1B.b). The gauze square was removed from a whirl-pak bag and gently scrubbed along the teat end and then placed back inside the bag. Teat canal samples were then collected by inserting a sterile cytobrush swab (cat. no. C0104, CooperSurgical Inc., Connecticut, USA) 5 mm inside the streak canal and rotating the swab 360° two times before removal (35) and placement inside a whirl-pak bag (Fig. 1B.c).

Stripped milk was collected prior to cisternal milk to avoid potential contamination (including blood) flowing from cisternal milk to stripped milk. Before the collection of milk samples, teat skin was prepped with iodine solution and 70% alcohol. After discarding the first three to four streams of milk, stripped milk samples (20 mL) were then collected in sterile falcon tube (Fig. 1B.d.) (23). The gland was then cleaned with an aseptic wash of iodine solution, and 70% alcohol and lidocaine was administered subcutaneously as local anesthetic. Cisternal milk samples were collected by puncturing the mammary gland cistern approximately 2 cm away from the teat base with a sterile 20-gauge collection needle, and the collected milk was released into two 10-mL vacuum collection tubes (Fig. 1B.a). Four sampling blanks were also collected as negative controls; “sampling blanks” (*N* = 2) consisted of gauze squares sealed in a whirl-pak bag and not opened until DNA extraction (Fig. 1B.f) and “air blanks” (*N* = 2) that consisted of gauze squares opened in the sampling location and waved in the air for 30 seconds but not used for actual sampling of the cows (Fig. 1B.e). Air sampling is notoriously difficult and technically complicated. Room-level air sampling (i.e., sampling all microbes in the milking parlor air) would require the use of powerful air sampling devices over extended periods of time. By contrast, our goal in this study was to obtain a snapshot of the airborne microbes in the direct vicinity of each cow at the time of sampling, since these microbes would be the most likely to contaminate our samples. All samples were placed inside a cooler during sampling and then brought immediately to the University of Minnesota Food Centric Corridor for further processing. Aliquots of 1 mL of stripped milk and 1 mL of cisternal milk were placed inside separate sterile vials in a biosafety cabinet and stored at −20°C until submission for microbiological culture (Fig. 1C). The remaining milk was stored at −80°C, along with the teat apex gauze, teat canal cytobrush, and sampling and air blank samples.

### Bacterial culture

Aliquots of stripped and cisternal milk stored at −20°C were submitted to the Udder Health Lab at the University of Minnesota for bacterial culture and taxonomic identification within 2 weeks of their original collection date according to a previous protocol (36). Briefly, milk samples were plated onto 5% sheep blood agar using a 0.01-mL calibrated loop and incubated in aerobic conditions at 37 ± 2°C for 42 to 48 h. Samples were classified as contaminated if more than two isolates from different bacterial taxa were recovered. Cultured isolates were used as input to a matrix assisted laser desorption ionization-time of flight (MALDI-TOF) mass spectrometer (Microflex, Bruker Daltonics Inc., Billerica, MA) for taxonomic identification (35). Peaks produced by each isolate were analyzed by the MALDI-TOF Biotyper reference library using the following confidence level: >2.0, species level classification recorded; 1.8–2, genus level classification recorded; and <1.8, MALDI-TOF diagnosis not recorded, and traditional identification methods, such as differential growth on selective media, colony morphology, and Gram stain, were used for taxonomic classification (37).

### Propidium monoazide treatment

All stripped and cisternal milk samples were subjected to propidium monoazide (PMA) dye treatment to separate DNA from viable and non-viable bacteria (16). In a 1.5-mL centrifuge tube, 10 µL of PMA solution (1 mg/mL) was added to 1 mL of milk sample for a final concentration of 10 µg/mL. All solutions were then incubated at room temperature in the dark with agitation for 5 minutes. Samples were then placed on ice and exposed to a 500-W halogen light (SMART electrician, Menards, St. Paul, MN) at a distance of 20 cm for 5 minutes with occasional inversions to ensure total exposure. The samples were then centrifuged at 12,000 rcf for 5 minutes, after which the supernatants were removed, and the pellets were washed three times with an equal volume of phosphate buffer solution (pH 7.4) to remove any excess PMA before being stored in −20°C until DNA extraction.

### DNA extraction, library preparation, and sequencing

All teat skin, teat canal, milk samples, PMA-treated milk samples, and all sampling blanks were subjected to DNA extraction using the PowerSoil Pro Kit (Cat No. 47016, Qiagen, Hilden, Germany), automated on the QiaCube Connect Instrument (Cat No. 9002864, Qiagen, Hilden, Germany). Briefly, whirl-pak bags containing teat apex gauze or teat canal cytobrush samples were thawed for 20 minutes in a biosafety cabinet sterilized using 70% EtOH. Once thawed, the gauze or the tip of the cytobrush was cut using a metal pair of scissors. The dirtiest part of the gauze or the cytobrush tip was placed inside a PowerBead Pro tube. This process was repeated for each teat apex and canal sample using metal forceps and scissors that were sterilized with a glass-bead sterilizer (250F). DNA extraction for milk samples proceeded according to the manufacturer’s instructions. The instrument was run six times, once for each batch of 12 samples that were processed. Each batch included an extraction blank control (“Extraction,” *N* = 6), containing 800 μL of CD1 lysis buffer in an empty bead beating tube without any sample material (Fig. 1 B.g). The purpose of the extraction blank control was to identify and account for reagent contamination from the DNA extraction kit. A single positive control sample (“Positive”), containing a known concentration and taxonomy of eight bacterial and two fungal species of intact cells, was also included as an internal control (Zymo Research Corp., Cat No. D6310). Extracted DNA from each sample was randomized into a 96-well rack (Fig. 1C), and approximately 20 μL of each sample was transferred from the rack to a PCR plate, covered with an adhesive seal, and then submitted to the University of Minnesota Genomics Core (UMGC) for library preparation and sequencing.

Extracted DNA was quantified and assessed for quality by both NanoDrop UV/VIS spectrophotometry (Thermo Fisher, USA) and fluorometry using PicoGreen staining (BioTek, USA). The 16S rRNA gene copy number in each sample was determined using qPCR. Libraries were prepared by amplifying the V4 region of the 16S ribosomal RNA gene, using primer Meta_V4_515F: GTGCCAGCMGCCGCGGTAA and Meta_V4_806R: GGACTACHVGGGTWTCTAAT (38). Negative controls for library preparation (“Library,” *N* = 5) were included during the library preparation process, consisting of molecular-grade water added to five randomly selected wells (Fig. 1B.h). Prepared libraries were sequenced to an expected sequencing depth of 100,000 paired-end reads per sample on an Illumina MiSeq instrument (Illumina Inc., San Diego, CA) using a 600 (2 × 300 base pair) cycle reagent kit (Illumina Inc, San Diego, CA).

### Bioinformatics and sequencing efficiency

All the following bioinformatic and statistical analyses were performed in R Statistical Software (v4.1.2; R Core Team 2021; https://www.r-project.org/), and plots were generated using the ggplot2 v3.4.2 package in R (39). Raw sequence data were processed through the DADA2 (v1.22.0) pipeline for quality filtering, denoising, and microbial community inference (40). The filterAndTrim function was used to quality filter the raw sequence data. Primer pairs were removed by trimming the first 20 and 17 base pairs from the 5′ ends of forward and reverse reads, respectively. Forward and reverse reads were truncated to a length of 270 and 200 base pairs based on the observed distribution of quality score, and sequence reads containing ambiguous base pairs and PhiX were discarded. Forward and reverse reads with a maximum expected error rate greater than 3 or 4 base pairs, respectively, were also discarded. The learnErrors function was used to estimate the expected error rates produced by the Illumina MiSeq sequencer. Error-corrected reads extending beyond or below the expected length of the sequenced amplicon were discarded. Forward and reverse reads were concatenated using the mergePairs function. Merged reads were used as input to the removeBimeraDenovo function to identify and remove chimeric sequences. The merged reads with a length beyond 250 and 258 base pairs were discarded based on the distribution of sequence reads and the length of the expected V4 region. Chimera-free amplicon sequence variants (ASVs) were aligned to the SILVA reference database (v138.1) for taxonomic assignment using the assignTaxonomy function. The ASV abundance matrix, taxonomy table and sample metadata were used to generate a phyloseq object for microbiome data analysis using the phyloseq (v1.38.0) R package (41).

The sequence data for the positive control sample were aligned to the manufacturer’s sequence database (https://s3.amazonaws.com/zymo-files/BioPool/ZymoBIOMICS.STD.refseq.v2.zip) containing 16S rRNA reference sequences for each mock bacterium, using the same procedures described above. Extraction and sequencing efficiency were measured by inspecting the distribution and taxonomy of the positive control sample. Sequence features classified to *Listeria*, *Pseudomonas*, *Bacillus*, *Escherichia*, *Salmonella*, *Lactobacillus*, *Enterococcus*, and *Staphylococcus* spp. were extracted, and their distribution was visually assessed via bar plots.

Due to the lack of classification for most reads in the data set of PMA-treated samples, all the following microbiome analyses were performed on the data set generated by samples without PMA treatment. Quality filtered sequence reads from PMA-treated samples were aligned to the *Bos taurus* reference genome (NCBI RefSeq assembly: GCF_002263795.2) using Kraken2 (42).

### Identification and removal of potential contaminants

Potential contaminants obtained during sampling, DNA extraction, and sequencing were identified using decontam (43) and SourceTracker (44) packages in series. First, the phyloseq object was subjected to the decontam package (v1.14.0) to identify potential sequence contaminants introduced during DNA extraction and library preparation. The isContaminant function was used to identify contaminants using both the frequency- and prevalence-based methods at the threshold of 0.5 after plotting the distribution of score statistics, as recommended by Davis et al. (43). Sequence features classified as potential contaminants using the frequency method were visualized by plotting the abundance of the contaminant within each sample as a function of that sample’s measured 16S qPCR concentration using the plot_frequecy function. The prevalence method was run two times, using extraction blanks and library preparation blanks as negative controls, since different contaminants were expected from each type of blank. ASVs identified as contaminants were then removed from the phyloseq object.

The contaminant-subtracted phyloseq object was then subjected to the SourceTracker package to identify the proportion of ASV counts in the animal samples (i.e., teat apex, teat canal, stripped milk, and cisternal milk) that could be attributed to negative controls. In this analysis, negative controls were defined as source environments and animal samples were defined as sink environments. The proportion of each individual ASV arising from each source was then predicted and removed from the animal samples according to previously reported methods (32). The reduced data set was then used for all subsequent microbiome analyses. The SourceTracker analysis was then used to understand the contribution of (i) the negative controls, teat apex, and canal (sources) to the cisternal milk (sink) and (ii) the negative controls, teat apex, canal and cisternal milk (sources) to the stripped milk (sink).

### Microbiome diversity analysis

Alpha diversity of true samples and negative controls was estimated by calculating richness, Shannon diversity index, and inverse Simpson’s index of diversity, using the estimate_richness function in the phyloseq package. Alpha diversity metrics stratified by sample types were visualized via raincloud plot using the gghalves (v0.1.4) package (45).

Beta diversity was estimated by computing pairwise Bray-Curtis distances between all animal samples and negative controls, and microbiome dissimilarities were visually compared by non-metric multidimensional scaling (NMDS) using the ordinate function in the phyloseq package. Ordination fit was assessed by increasing the “trymax” parameter, i.e., the maximum number of random starts used to search for a stable solution, until stress value < 0.2 was obtained. The proportion of variation in the microbiome that was due to the sample type was estimated using a permutational multivariate analysis of variance (PERMANOVA) (46). The pairwise.adonis2 function implemented in the vegan package was used after 999 permutations (47). The homogeneity of variance between sample types was estimated by the betadisper function implemented in the vegan package and analysis of variance (ANOVA).

### Potential mastitis pathogens

To evaluate the prevalence of potential mastitis pathogens in the animal samples, we subsetted the genus-level count matrix of the 16S rRNA data to only include the potential pathogen candidates reported in a previous study (48). Here, the count matrix was the clean data set after running decontam and SourceTracker for decontamination.

### Statistical analysis

To describe the difference in sequencing depth, 16S gene copies (log10), and alpha diversity indices between different sample types, a generalized linear model was fit using the glm function. Model significance was assessed using ANOVA. Adjusted means were computed between sample types using the emmeans package (49), with significance being determined at the 0.05 level.

## RESULTS

### Bacterial culture

Cisternal and stripped milk samples were subjected to standard microbiological culture typically used for mastitis testing (Table 1). Among cisternal samples, one sample exhibited growth of *Staphylococcus chromogenes*, while the remaining 13 exhibited no bacterial growth. Among stripped milk samples, seven exhibited no bacterial growth, three exhibited growth of *Corynebacterium* sp., two exhibited growth of Gram-negative bacteria, one exhibited growth of *Staphylococcus chromogenes*, and two were contaminated (i.e., growth of three or more bacteria). The cisternal milk sample with growth of *Staphylococcus chromogenes* was collected from one of the cows (ID, 3019) whose stripped milk sample also grew *Staphylococcus chromogenes* and was classified as contaminated due to the growth of more than two microorganisms.

**TABLE 1:** Number (%) of samples containing zero, one, or two distinct microorganisms, stratified by the type of milk sample collected (cisternal versus stripped)^*a*^

	Zero	One	Two	Three or more (contaminated)	Microorganism name (CFU/0.01 mL^*b*^)
Cisternal milk	13/14 (92.9%)	1/14 (7.1%)	0 (0%)	0 (0%)	*Staphylococcus chromogenes* (1^*b*^)
Stripped milk	7 (50.0%)	4 (28.6%)	1 (7.1%)	2 (14.3%)	*Corynebacterium* sp. (4^*b*^), *Staphylococcus chromogenes* (5^*b*^), *Corynebacterium* sp. (1^*b*^), Gram-negative organism (1^*b*^)

^*a*^ The corresponding cow ID can be found in Table S1.

^*b*^ The number in parentheses following the microorganism name represents the number of colonies recovered from 0.01 mL of milk.

### Sequencing depth and quality

**Fig 2:**
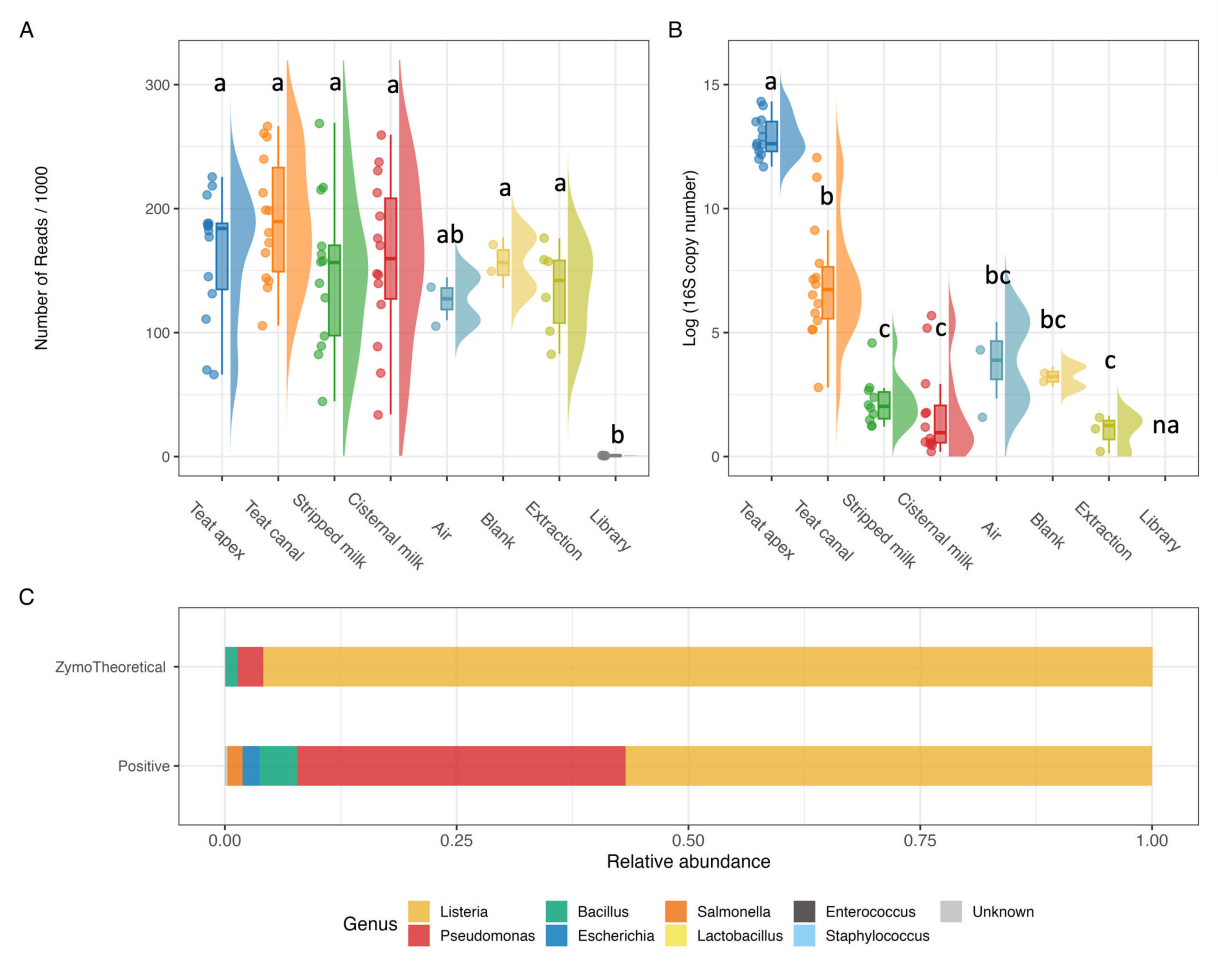
(**A**) Sequencing depth and (**B**) 16S gene copy number across sample types, including animal samples and negative controls. (**C**) Relative abundance of genus composition in positive control sample and Zymo Theoretical (theoretical standard) samples; the category “Unknown” represents ASVs that were not classified as any of the eight bacterial/archaeal taxa contained within the mock community. Different letters within each panel indicate significant differences between sample types. na = not applicable.

In total, 10.8 M raw paired-end reads was generated from 72 samples, including animal samples and negative controls, with an average of 149,654 reads per sample (range: 563–269,313). A total of 8.5 M remained after quality filtering and merging the forward and reverse sequence reads, and 6.9 M paired-end sequence reads remained after removing chimeras. One stripped milk sample (cow ID: 4778) was removed from further analysis because it contained a lower number of reads than the extraction blanks, suggesting a failed library. After removing this outlier sample, no significant differences in sequencing depth were observed among sample types, including animal samples (i.e., teat apex, teat canal, stripped milk, and cisternal milk), sampling controls (i.e., air and blank), and extraction controls (Fig. 2A, ANOVA, *P* > 0.05). Library controls showed a significantly (ANOVA, *P* < 0.05) lower number of reads than the other samples, except for air controls.

The log-transformed 16S copy number was significantly higher in teat apex samples than all other sample types (ANOVA, *P* < 0.05), followed by teat canal samples, which were similar to sampling controls (i.e., air and blank) (Fig. 2B). Stripped milk and cisternal milk samples had the lowest 16S copy number among animal samples, and these values were not significantly different from sampling and extraction controls (Fig. 2B).

The positive control sample generated 235K raw sequences, and the 186K sequences that remained after quality control were assigned to 34 taxa using the Zymo reference database (Fig. 2C; Table S2). Six of these 34 taxa were from the mock community, while the rest were unidentified but accounted for 0.2% of the total sequences generated from the mock community sample. *Escherichia* and *Staphylococcus* (theoretical abundances of 0.061 and 0.01%, respectively) were not recovered in the positive control sequence data, possibly due to their low abundance within the mock community. In addition, *Pseudomonas* was overrepresented with 35.4% of the sequence data (theoretical relative abundance 2.8%), while *Lactobacillus* was underrepresented at 56.8% (theoretical relative abundance 95.9%). These results indicate deviations between our observed microbial community and the theoretical composition of the positive control and indicate our sequencing might be biased toward the microbial profile of our collected samples.

### Decontamination

**Fig 3:**
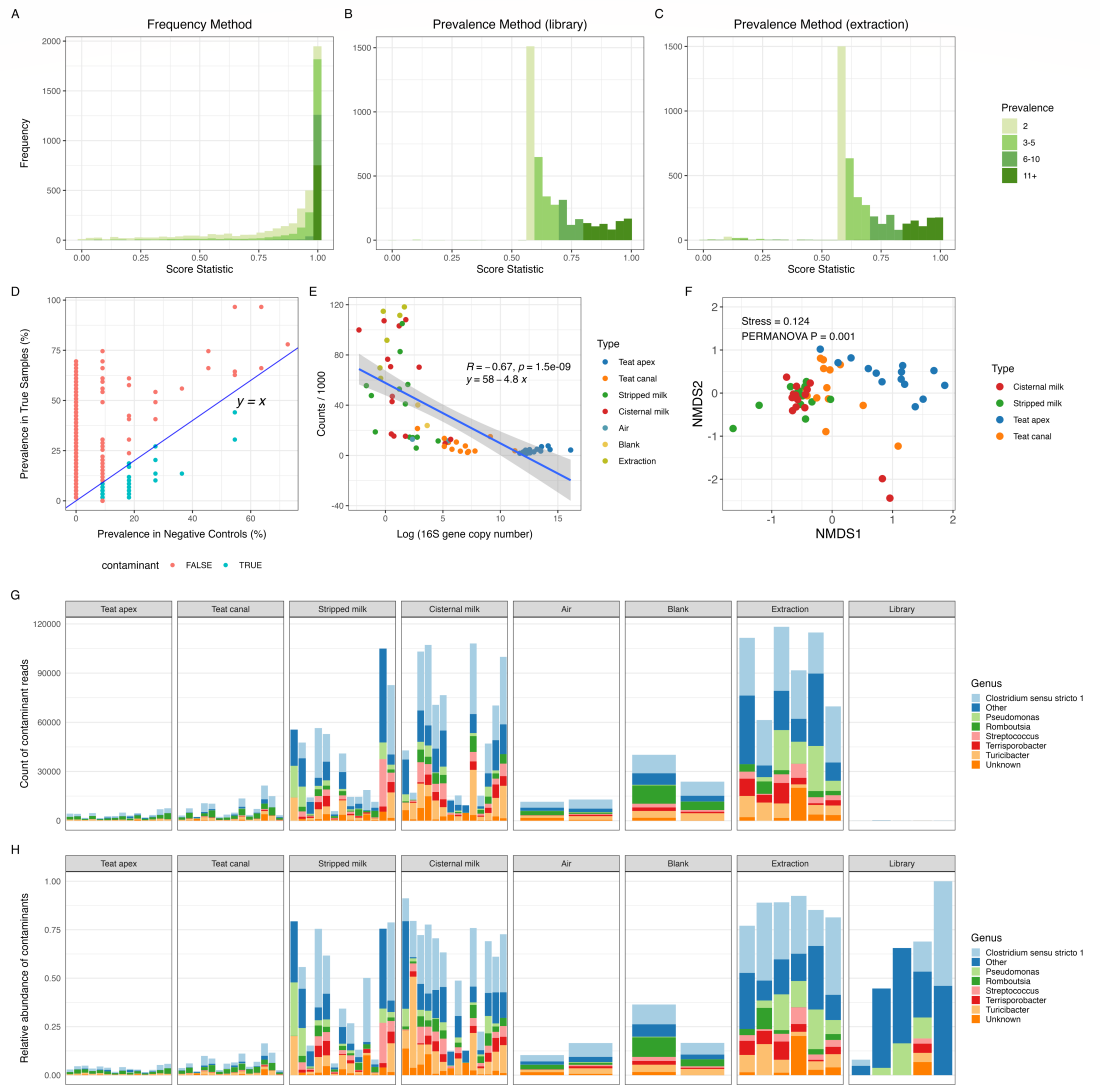
Contaminants identified using decontam (43). Histograms showing the distribution of score statistics assigned to ASV present in two or more samples using (**A**) frequency method, (**B**) prevalence method with library blanks as negative control, and (**C**) prevalence method with extraction blanks as negative control. Color intensity indicates the number of samples in which each ASV was present (i.e., prevalence). (**D**) Prevalence (%) of contaminants (teal, “TRUE”) and non-contaminants (pink, “FALSE”) identified by the prevalence method in true animal samples (*y*-axis) and negative controls (*x*-axis); (**E**) correlation between 16S copy number (*x*-axis) and counts of contaminants/1,000 (*y*-axis) from each sample identified by the combined frequency and prevalence method; (**F**) NMDS ordination of Bray-Curtis distances of decontam contaminants identified from milk, teat apex, and teat canal samples; (**G**) genus-level counts of contaminant reads identified by combined frequency and prevalence methods; genera with relative abundance < 5% and prevalence < 20% across all samples were grouped into “Other;” (**H**) relative abundance of genus-level contaminant reads identified by combined frequency and prevalence methods; genera with relative abundance < 5% and prevalence < 20% across all samples were grouped into “Other;” see File S2 for the full list of taxa names and their abundances.

Decontamination was first performed using decontam by both prevalence and frequency methods (Fig. 3). The score statistic assigned by decontam was used to classify sequence features (ASVs), with a score < 0.5 indicating a contaminant. Using the frequency method, the majority of sequencing features (prevalence > 2) were assigned scores close to 1.0, suggesting that they were not contaminants (Fig. 3A). Using the prevalence method, we observed a unimodal distribution of scores around 0.5 using either library blanks (Fig. 3B) or extraction blanks (Fig. 3C) as negative controls. The contaminants identified by the prevalence method, using both library and extraction as negative controls, confirmed that true contaminants (denoted in teal, under the diagonal line) had higher prevalence in negative controls than in true samples (Fig. 3D).

The frequency method identified 422 contaminant ASVs, while the prevalence method identified 11 and 111 contaminant ASVs using library blanks and extraction blanks as negative controls, respectively. In total, 516 ASVs representing 2.1 M reads were identified as contaminants using both methods, which accounted for 31.9% of the filtered reads. Among the sample types, extraction blanks contained the highest abundance of contaminants, followed by cisternal milk and stripped milk (Fig. 3E and G). Teat apex and teat canal samples contained a lower abundance of contaminants compared with sampling controls (i.e., air and blank). The number of reads originating from contaminant ASVs was inversely correlated with the 16S gene copy number, suggesting that low microbial biomass samples were prone to microbial contamination during sample collection and processing (Fig. 3E and H). The structure of sequence features within the contaminant data showed significant differences between sample types (Fig. 3F, PERMANOVA, *P* = 0.001, *R*^2^ = 0.32). Only the contaminants identified in stripped milk and cisternal milk showed no significant differences in microbial composition based on beta-diversity analysis, suggesting that the profile of contaminants in these samples was similar (Table S3, PERMANOVA, *P* = 0.200, *R*^2^ = 0.049). Though a small amount of contaminants was identified in library controls (Fig. 3G, counts), most reads generated in library controls were still identified as contaminants (Fig. 3H, relative abundance). *Clostridium sensu stricto 1* was dominant among identified contaminants across all sample types, while *Pseudomonas*, *Romboutsia*, *Streptococcus*, and *Turicibacter* also showed relatively high abundance (abundance > 5%) and prevalence (>20%) among the contaminant data (Fig. 3G and H). All identified contaminant reads were removed from the data set, and the remaining reads were subjected to SourceTracker to identify the proportions of contribution from defined sources.

**Fig 4:**
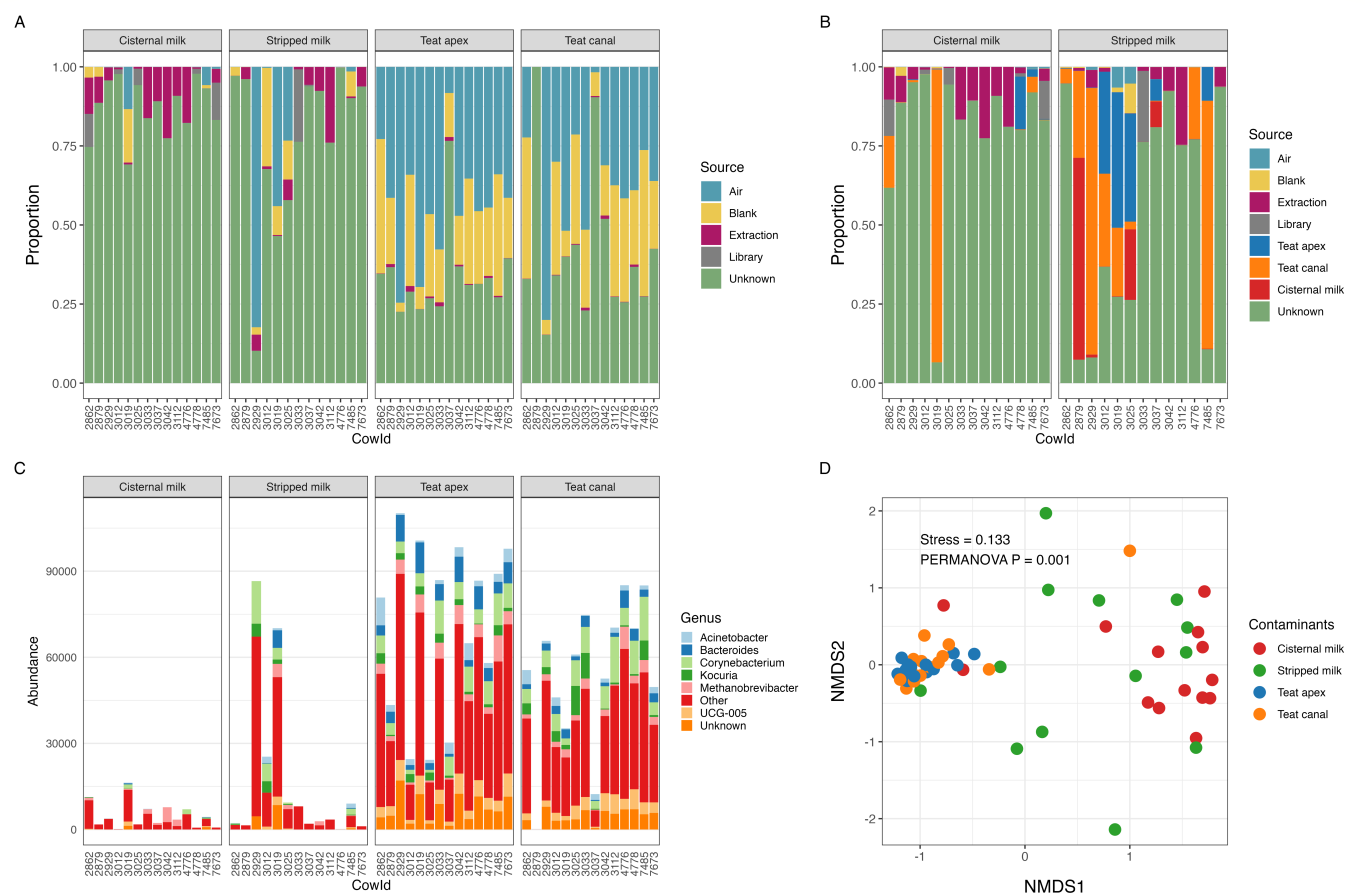
Source of contaminants identified by SourceTracker. (**A**) Proportion of reads identified as contaminants using negative controls (i.e., air, blank, extraction, and library) as sources; (**B**) proportion of reads identified as contaminants using negative controls, teat apex, and teat canal as sources; (**C**) counts of genus-level contaminant reads using negative controls as source environments; genera with relative abundance < 5% and prevalence < 25% across all samples were grouped into “Other;” see File S2 for the full list of taxa names and their abundances; (**D**) NMDS ordination of Bray-Curtis distances of SourceTracker contaminants identified in milk, teat apex, and teat canal samples.

After running decontam, the proportion of reads in each sample originating from negative controls (i.e., air, blank, extraction, and library) was calculated using SourceTracker (Fig. 4A). A large proportion of reads within the samples from the teat apex (66.2%) and teat canal (57.8%) was sourced from negative controls (primarily sampling and air blanks), while these sources represented only a small proportion of the reads in the cisternal milk and stripped milk samples (13.0% and 23.3%, respectively; Fig. 4A). This indicated that the environment may be an important source of contamination (both from the farm and laboratory environment) for teat skin samples. Library blanks showed little contribution to any of the milk, teat apex, or teat canal samples suggesting that the library preparation process introduced little contamination to true samples (Fig. 4A). Across all the cisternal milk samples, the main sources of contaminating reads were from extraction blanks (7.6%) but rarely from air blanks (1.5%) and sampling blanks (1.8%). This differed from stripped milk samples where air blanks comprised the highest source of contamination, followed by sampling blanks and extraction blanks (11.7%, 5.1% and 4.7%, respectively). It is notable that the contamination sources for the milk samples were highly variable across individual samples (Fig. 4A and B), such that each sample seemed to have its own dominant contamination source. This highlights the unpredictability in defining sources that contribute to the microbiome data generated from bovine milk samples. On the other hand, teat apex and teat canal showed a consistent pattern of contamination sources, which were mainly air blanks (mean = 38.3%, SD = 19.1%) and sampling blanks (mean = 23.2%, SD = 13.2%). Most of these identified contaminants were from genera *Corynebacterium*, *Acinetobacter*, *Bacteroides*, *UCG-005*, *Kocuria*, and *Methanobrevibacter* (Fig. 4C). The sample type had a significant effect on the microbial composition of sourced contaminants identified by SourceTracker (Fig. 4D, PERMANOVA *P* = 0.001, *R*^2^ = 0.24). However, there were no significant differences in the microbial community of contaminants between stripped and cisternal milk samples (PERMANOVA *P* = 0.222, *R*^2^ = 0.048) or between teat apex and teat canal samples (PERMANOVA *P* = 0.353, *R*^2^ = 0.039) (Table S4). When teat apex and teat canal samples were included in potential sources of contaminants in the milk samples, SourceTracker indicated that a majority of the stripped milk samples contained a high proportion of sequences from the teat apex and teat canal, while only one of the cisternal milk samples (cow ID, 3019) was dominated by contaminants originating from the teat canal (Fig. 4B).

### Microbial diversity

**Fig 5:**
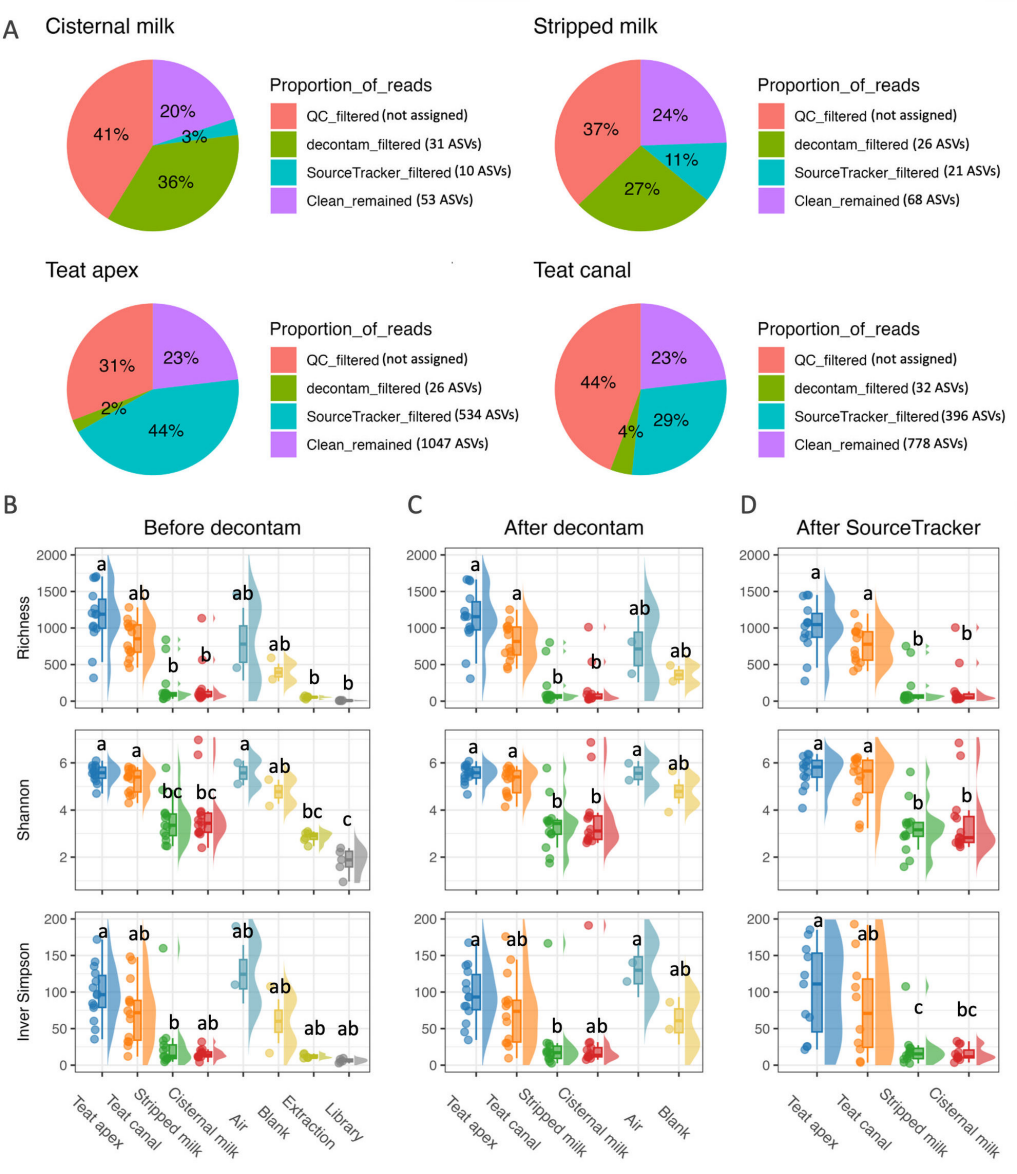
Alpha diversity before and after decontamination with decontam and SourceTracker. (**A**) Pie charts showing the percentage of reads that were removed during quality control (“QC,” low-quality reads), “decontam,” and “SourceTracker” as well as the percentage of remaining reads at the end of the decontamination process (“Clean”), stratified by sample type. For each step of the decontamination process, the number in the parentheses indicates the median number of ASVs removed by decontam and SourceTracker and the median number of ASVs that remained in the “Clean” reads. Raincloud plots for alpha diversity indices (i.e., richness, Shannon’s, and Inverse Simpson’s) (**B**) before decontam, (**C**) after removing contaminants identified by decontam, and (**D**) after removing contaminants identified by SourceTracker, stratified by sample type. Sample groups with different superscripts had significantly different alpha diversity values within each facet B–D.

We next assessed how quality filtering, decontam, and SourceTracker impacted alpha diversity metrics. Across all samples, a relatively high proportion of sequencing reads was removed during the QC process (Fig. 5A). After removal of putative contaminants by both decontam and SourceTracker, the samples within each sample type retained only ~25% of the total reads generated. Identification of contaminants differed by sample type, with decontam identifying most of the contaminants in cisternal and stripped milk samples (36% and 27%, respectively) and SourceTracker identifying most of the contaminants in the teat apex and teat canal samples (44% and 29%, respectively; Fig. 5A; Fig. S1). Reads identified as contaminants by decontam represented a fairly consistent number of unique ASVs (*N* = 26–32) across sample types. In contrast, reads identified as contaminants by SourceTracker belonged to a very large number of unique ASVs within the teat apex and teat canal samples (534 and 396, respectively) and a much smaller number of unique ASVs in the cisternal and stripped milk samples (10 and 21, respectively). After decontamination, the numbers of remaining reads (95% CI) were 37.8K (24.4–51.1K), 44.3K (24.4–64.2K), 36.3K (21.4–51.3K), and 31.7K (19.6–43.9K) for teat apex, teat canal, stripped milk, and cisternal milk samples, respectively (Table S5).

Patterns of alpha diversity (as measured by microbial richness, Shannon diversity, and Inverse Simpson) remained relatively stable across the decontamination process (Fig. 5B through D). At each step of the decontamination process, the order of diversity (from highest to lowest) was teat apex > teat canal > air > blank > stripped milk > cisternal milk > extraction > library. In all cases, the teat canal and teat apex samples showed significantly higher richness and Shannon’s index than cisternal and stripped milk samples. However, across all alpha diversity indexes, teat apex samples did not have significantly different diversity than teat canal samples, and the diversity of both teat apex and teat canal samples was similar to that of the sampling controls (i.e., air and sampling blanks). Similarly, stripped milk and cisternal milk had no significant differences in all alpha diversity indices across all steps of the decontamination process, and their diversity values were similar to that of extraction and library blank controls. Thus, relative ranking of sample types by their alpha diversity index was teat apex = teat canal = air = blank > stripped milk = cisternal milk = extraction = library.

**Fig 6:**
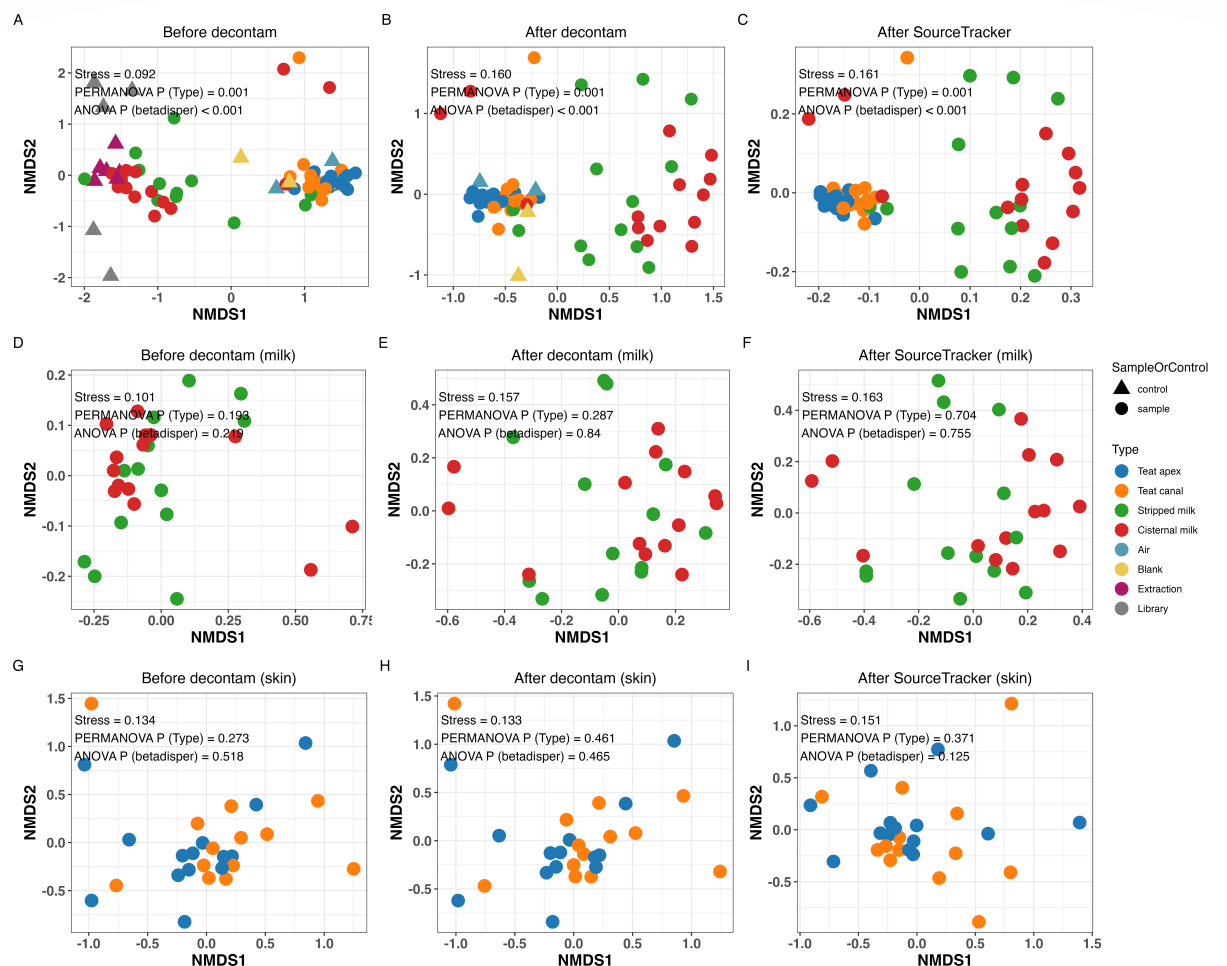
NMDS ordination based on Bray-Curtis distance matrix, using the entire data sets (**A**) before decontam, (**B**) after decontam, and (**C**) after SourceTracker, (**D–F**) using the milk samples data sets, (**G–I**) using teat apex skin and teat canal sample data sets. PERMANOVA *P* is the *P* value for sample types; ANOVA *P* is the *P* value for betadisper variance between sample types.

NMDS ordination and PERMANOVA were performed to visualize and compare the composition of the microbial communities between different data sets (i.e., before decontam, after decontam, and after SourceTracker) and between sample types (i.e., teat apex, teat canal, cisternal milk, and stripped milk) (Fig. S2). Results showed that microbial composition at the ASV level varies within the overall data set and within sample type (PERMANOVA *P* < 0.001 in both cases). Hence, the comparison of microbial community composition was performed on data sets stratified by decontamination step. Subsequent NMDS analysis showed that the microbial composition clustered according to sample type, and this clustering pattern was maintained across the decontamination process (Fig. 6A through C). Air and blank controls cluster closely with teat apex and canal samples, while the extraction and library blanks cluster closely to the stripped and cisternal milk samples (Fig. 6A). However, milk samples displayed significantly higher beta-dispersion than teat apex and teat canal samples (ANOVA *P* < 0.01), and thus, we also stratified by sample type (Fig. 6D through I). At each step of the decontamination process, no significant differences in beta diversity were observed between teat apex and teat canal samples or between milk samples (stripped milk vs cisternal milk) (Fig. 6D through I; Table S6). However, teat apex and canal samples and milk samples displayed significant differences in the microbial composition across the decontamination process.

### Microbial composition

**Fig 7:**
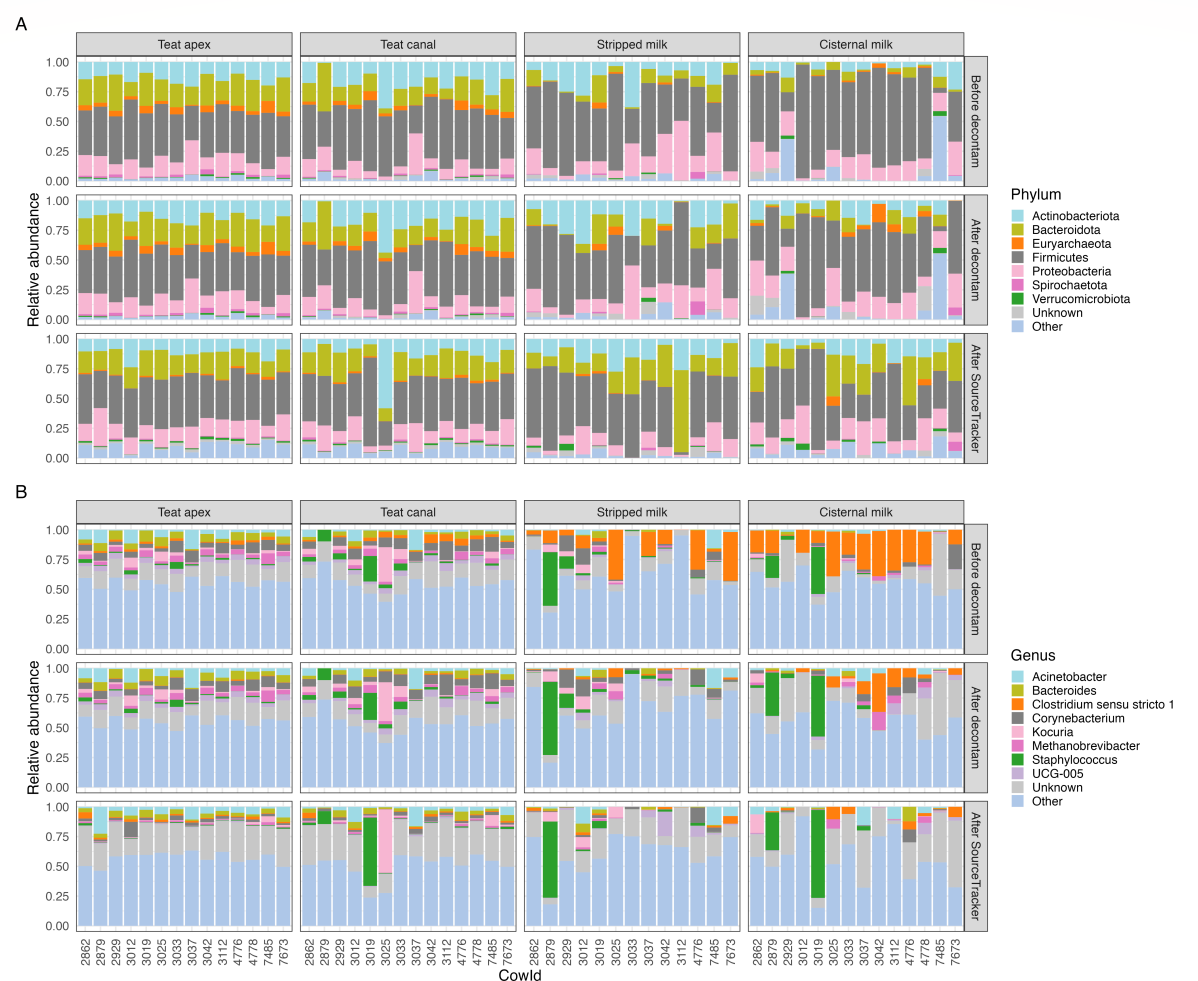
The relative abundance of most abundant taxa in animal samples detected before decontam (top row), after decontam (middle row), and after SourceTracker (bottom row) at (**A**) phylum level (abundance < 1% and prevalence < 20% were grouped as “Other”) and (**B**) genus level (abundance < 3% and prevalence < 20% were grouped as “Other”); see File S2 for the full list of taxa names and their abundances.

The microbial composition and relative abundance of top taxa from skin and milk samples shifted across the decontamination process, at both the phylum (Fig. 7A) and genus (Fig. 7B) levels. After use of SourceTracker, Firmicutes was the dominant phyla across all skin and milk samples, accounting for 41.9% of sequence counts (Fig. 7A). Bacteroidota (19.9%), Proteobacteria (13.9%), and Actinobacteriota (13.0%) were also predominant. At the genus level, no clearly predominant microorganisms were observed in either skin or milk samples (Fig. 7B), which exhibited remarkable consistency in microbial profiles, except for a few samples with a high relative abundance of *Staphylococcus*. After decontamination, *Staphylococcus* (4.9%) and *Acinetobacter* (4.2%) were the top two genera in relative abundance across all samples. Among the top genera, *Acinetobacter*, *Bacteroides*, and *Corynebacterium* exhibited higher abundance in skin versus milk samples, while *Clostridium sensu stricto 1* showed the opposite trend. The relative abundance of *Clostridium sensu stricto 1* decreased substantially during the decontamination process, especially in milk samples, demonstrating its importance as a major source of contaminating microbial DNA. *Staphylococcus* showed high relative abundance in cisternal milk (7.9%), stripped milk (5.9%), and teat canal (5.5%) samples but lower relative abundance in teat apex (0.6%) samples.

### Presence of potential mastitis pathogens

**Fig 8:**
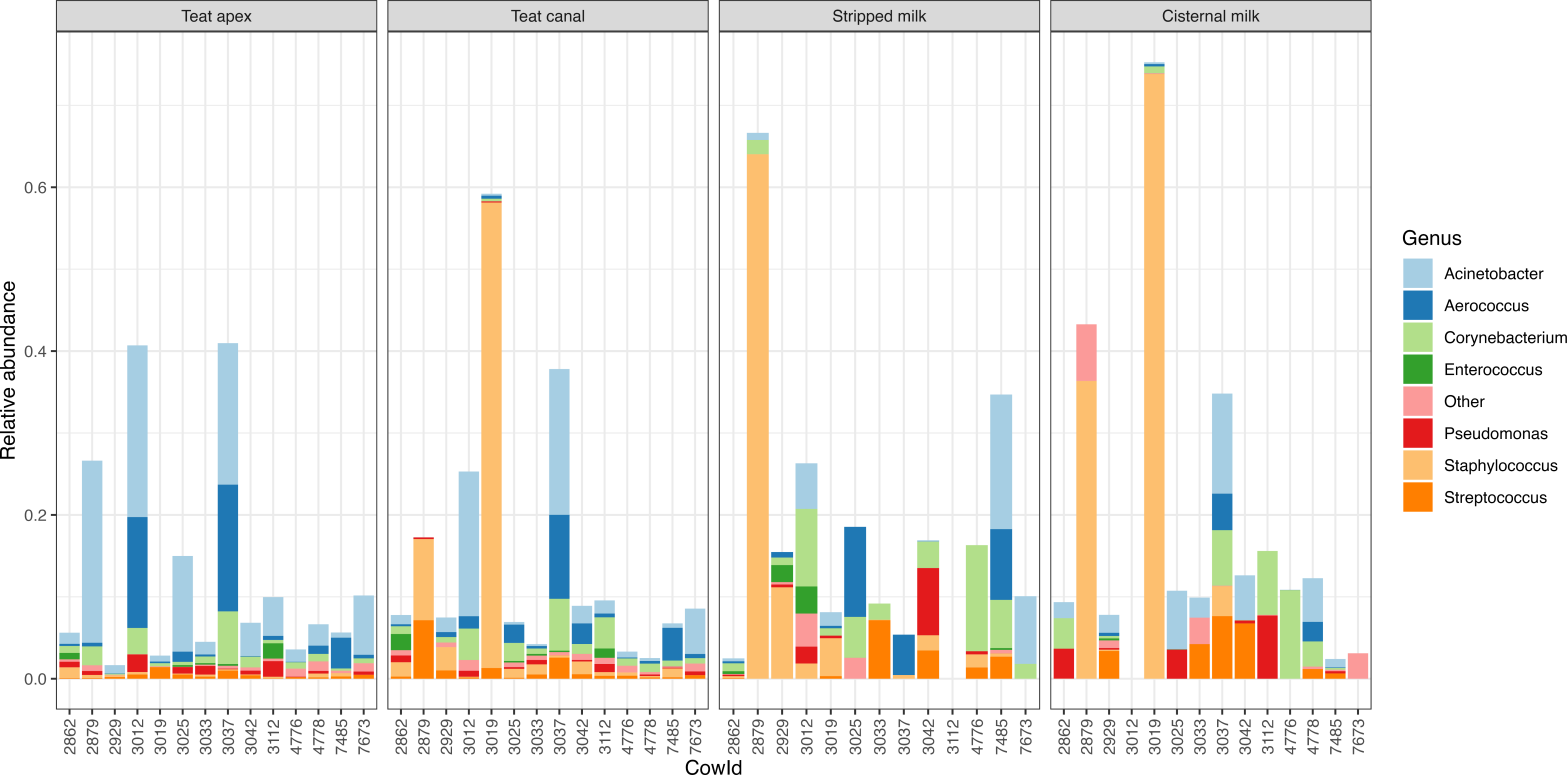
Barplot of the relative abundance (*y*-axis) of potential mastitis pathogens (proportion of all genus-level counts) in each sample (*x*-axis) after decontam and SourceTracker, grouped by sample type. Only genera with a relative abundance > 0.1% and prevalence > 25% are depicted as individual colors within the bars; the rest are grouped together as “Other;” see File S2 for the full list of taxa names and their abundances.

The presence of potential mastitis pathogens in relative abundance across all samples is shown in Fig. 8. On average, sequences from potential mastitis pathogens accounted for 14.8% of the reads across all sample types. At the genus level, the potential pathogens with the highest relative abundance were *Staphylococcus* (5.1%), *Acinetobacter* (4.0%), *Corynebacterium* (2.2%), *Aerococcus* (1.7%), *Streptococcus* (1.1%), and *Pseudomonas* (0.7%). Samples from two cows (Cow IDs, 2879 and 3019) had distinctly greater abundance of *Staphylococcus* in their cisternal milk, stripped milk, and teat canal samples (Fig. 8). Interestingly, the milk samples from these two cows also yielded growth of *Staphylococcus* during bacterial testing (Table 1). *Streptococcus* sequences also showed high relative abundance in cisternal milk (1.7%), stripped milk (1.2%), and teat canal (1.1%) samples but only 0.4% relative abundance in teat apex samples. Teat apex contained a high relative abundance of *Acinetobacter* (7.0%) when compared with other sample types.

### Microbial composition of PMA-treated samples

Sequencing of samples treated with PMA (*N* = 60) generated 1.9 M raw paired-end reads, and only 0.3 M reads remained after removing chimeras. Among these non-chimeric reads, 18.9% was classified as Proteobacteria and 1.58% was assigned to Cyanobacteria at the phylum level (Fig. S3A). At the family level, only 7.1% of the non-chimeric reads was classified, and all of them were classified as *Mitochondria* (Fig. S3B). Zero non-chimeric reads were classified at the genus level (data not shown). Due to the lack of classification for most reads in the data set of PMA-treated samples, we performed additional analysis to align these reads to the host genome. These results showed that an average of 83.6% of reads per stripped milk sample (range 6.4%–99.7% per sample) and 71.6% of reads per cisternal milk sample (range 0%–99.7% per sample) were classified as host reads. This indicates additional refinement is needed before this approach is suitable for bovine milk molecular diagnostics. We note here that even the samples from the two cows with culturable *Staphylococcus* did not yield classifiable sequencing reads in the PMA-treated samples data set.

## DISCUSSION

Contamination is ubiquitous in microbiome studies and is especially problematic for samples with low microbial biomass such as bovine milk. In this study, we aimed to characterize the “true” dairy cow milk microbiome from different compartments of the mammary gland by identifying potential contamination of milk from the external and internal teat epithelial microbiomes and from the sample handling process including sample collection, DNA extraction, and library preparation. Using multiple sampling controls and a two-step sequential statistical algorithm approach, we found that the majority of sequence reads was identified as contaminants and thus removed from microbiome analysis. After this contaminant identification and removal process, only 20%–24% of the reads remained for microbiome analysis, suggesting that the vast majority of sequence data typically generated from bovine milk and udder samples may represent contaminating microbes and/or DNA. This finding is important both for interpreting previous milk microbiome studies and for designing future studies. Previous results that do not incorporate extensive use of negative controls should be interpreted cautiously, because the majority of the data used in those studies could represent contamination. For future studies, it is important to include numerous negative controls so that the impact of contamination can be quantified and fully appreciated in interpreting results.

The contaminants that we identified in the milk samples mainly originated from the DNA extraction procedure (sometimes called the “kitome”). Contaminants of skin samples were mostly consistent with those identified in the sampling devices which indicates these contaminants were primarily from microbes in the air of the milking parlor and laboratory and from gloves worn during handling the devices. After removing all potential contaminant sequences, the milk microbiome profiles were less diverse, more dispersed, and compositionally distinct as compared with the skin microbiome profiles. However, our results indicate that a portion of the stripped milk microbiome might originate from microbes that inhabit the teat apex and teat canal. Even after the statistical decontamination procedures, potential mastitis pathogens were detected in both milk and skin samples and with relatively high abundance were detected in milk from cows that exhibited no signs of clinical mastitis, which has the potential of developing mastitis.

### Potential sources of contamination in bovine milk microbiome data

Our results suggest that contamination events occurred across the course of collecting and processing the bovine skin and milk samples. Blank controls, including sampling blanks, air blanks, and extraction blanks, generated 16S gene copy numbers that were similar to milk samples, highlighting the extremely low microbial biomass of milk from clinically healthy lactating cows and the subsequent challenge in differentiating true from contaminating DNA.

Milk samples (cisternal and stripped milk) generated very low DNA concentration in this study (Fig. 2B), which are consistent with general characterization of milk as having a low bacterial biomass with previous reports from cow milk (50, 51). This low DNA content is due in part to several anatomical, physiological, and immunological factors that can help minimize microbial presence within the gland, although microbes can invade the gland and cows do experience mastitis. These factors can also contribute to the presence of large amounts of host somatic cells in milk (16), which can make it challenging to separate DNA from host and microbial sources. Milk is also a technically challenging matrix for microbiome work because its complex chemical composition includes DNase and RNase and other components that can hamper PCR amplification and nucleic acid extraction (14, 16, 52).

In microbiome research, low microbial biomass samples are especially sensitive to reagent contamination (18, 32), which helps explaining the negative correlation we observed between sample microbial biomass (i.e., 16S qPCR copy number) and the abundance of contaminants identified by decontam (Fig. 3E). Our results from both decontam and SourceTracker suggest that contamination of cisternal milk samples mainly occurred during the extraction process (Fig. 3C and 4A) and support the presence of bacterial DNA within the extraction kit and PCR reagents. This has been described as the “kitome” effect (29, 53, 54). Similar conclusions about the impact of extraction have been reported for human milk (28).

The sources of contaminants for stripped milk were more complex than cisternal milk and included extraction blanks, teat apex, teat canal, and cisternal milk samples (Fig. 4B). This result is consistent with previous studies (33, 55), which suggest that teat apex skin is a source of the microbial population in cow milk. Our results expand on the potential sources of the cow milk microbiome by including technical blanks, air, and sampling blanks. During milk sampling and processing, epithelial bacteria can become dislodged and enter the milk being sampled, microbes in the air of the farm or the lab can contaminate the sampling device and/or the milk sample itself, and molecular reagents can contaminate the sample during processing. We note here that the transfer of epithelial and milk-borne bacteria within the teat canal or at the teat apex could be a bidirectional process, particularly if milk leakage is occurring or cows were recently milked. In our sampling scenario, it is impossible to determine the directionality of bacterial transfer events. By defining the skin samples as the source and milk samples as the sink, we are forcing directionality into the analysis and assuming that most of the bacterial transfer is from the skin to the milk. This assumption is partially supported by the fact that cisternal milk did not have the same SourceTracker profile as stripped milk, suggesting that cisternal milk is contaminated as it passes down through the teat canal and out of the teat apex. Sources of this contamination include bacteria within the teat canal, at the teat apex, and in the milking parlor environment. To definitively answer this question, additional studies are needed.

It is important to note the very high sample-to-sample variability in the contamination source profile identified by SourceTracker (Fig. 4A and B). This high amount of inter-sample variability is typical for low microbial biomass samples and demonstrates the difficulty in measuring the true milk microbiome of lactating dairy cows. We hypothesize that the large variation in the source(s) of milk microbes may be due to the stochastic nature of milk sampling and processing. During these activities, presumably sterile milk can be contaminated with bacteria from the numerous sources we have described in this work. The inherently low bacterial biomass in the milk exacerbates the impact of these events because the bacterial cells involved in a single contamination event can easily overwhelm the number of cells present in the original milk sample. Thus, these mixing and contamination events can easily increase sample-to-sample variability in the profile of source contamination. Library controls were expected to contain very low microbial biomass and indeed contributed little to the profile of milk and skin sample microbiomes. This only indicates samples are less likely to be contaminated during library preparation but that much of the source of “kitome” contamination occurs prior to library preparation.

### Characterization of the de-contaminated milk microbiome

Even after removal of potential contaminants using both decontam and SourceTracker, the milk samples continued to harbor microbial DNA. Indeed, 53 ASVs remained in cisternal milk and 68 ASVs in stripped milk, though their alpha diversity indexes were lower than those from the skin samples. Stripped milk had similar microbial composition as cisternal milk, which agrees with a previous study that found no significant differences in the microbiota of stripped milk and cisternal milk (24). Given that stripped milk should represent milk that was removed from the cistern at the time of sampling, we would expect the profiles of cisternal and stripped milk to be similar, particularly after contaminants from the skin and milk parlor air are removed. However, our finding could also stem from a type II error due to our relatively low sample size (*N* = 13 to 14 per sample type) and large variation observed between individual animals. Larger studies will be required to confirm whether cisternal milk and stripped milk microbiota are truly indistinguishable from one another after statistical decontamination. In addition, future studies might examine the correlation between clean read counts by taxa and quarter SCC values, considering the relationship between milk microbiome and inflammation as evidenced by SCC (7).

The profile of the decontaminated milk microbiome data in this study showed that *Staphylococcus* and *Acinetobacter* were the predominant taxa, which is in line with previous studies (56–58). However, *Bacteroidetes*, *Corynebacterium*, *Pseudomonas*, and *Staphylococcus* might be overestimated in previous bovine milk microbiome studies (4, 17, 24, 25, 56, 59), as they were identified as contaminants in our study. This highlights the importance of contamination removal for correct interpretation of milk microbiome data.

It is also important to note that the 16S sequencing data we analyzed represents DNA from both viable and non-viable bacterial cells. We attempted to discriminate between live and dead bacteria within the milk microbiome data by PMA incorporation into DNA from non-variable bacteria, but the data set from the PMA-treated samples contained no classifiable bacterial sequence reads. This could indicate that much of the bacterial DNA in the milk microbiome data originated from non-viable bacterial cells but our culture-based results indicated that 8 of the 28 milk samples did indeed contain viable *Staphylococcus* and *Corynebacterium* bacteria (Table 1). This inability to detect the presence of these bacteria in the PMA data supports previous reports that the PMA assay might not be applicable for milk samples (16). Moreover, the host DNA identified in the PMA-treated milk samples suggested that 16S primers bind to off-target DNA (e.g., host DNA) if there’s very little template and a ton of off-target DNA. This is in line with microbiome studies that investigate samples with very low microbial biomass and abundant host DNA (60).

### Potential sources of contamination in bovine teat skin microbiome data

The skin samples (i.e., teat canal and teat apex) exhibited distinct patterns of contamination compared with milk samples, as evidenced by decontam and SourceTracker (Fig. 5A). Results of SourceTracker revealed that the skin samples were mainly contaminated by microbes associated with the sampling devices (as represented by air and sampling blanks; Fig. 4A). These blanks were meant to represent all the environmental (i.e., airborne) and physical contact events that sampling devices are exposed to during sample collection and processing. For air blanks (which were opened and waved in the air near the cows), this would include the air in the milking parlor and in the laboratory and biosafety cabinet during sample processing. It would also include physical handling of the device during both sample collection and processing. For the sampling blanks (which were brought to the farm but not opened), these exposure events consisted of the air in the laboratory and biosafety cabinet and handling of the device during sample processing (but not during sample collection). Our study demonstrated that these air and sampling blanks contributed more than 50% of the reads that were generated from teat apex and teat canal samples (Fig. 4A), highlighting the importance of aseptic techniques and the inclusion of negative controls during sample collection and processing. To be noted, we were not able to avoid milk contamination to the teat canal samples, because basically, all the cytobrushes were somewhat wet by milk after sampling. However, the microbial biomass of the teat canal was much larger than the milk as we observed in our study (Fig. 2B), which minimizes the contamination from milk samples to teat canal samples. Future analysis could set milk as a source and teat canal or teat apex as sink to identify the potential milk contaminations to skin samples.

It is important to note here that the environmental microbiome on a dairy farm likely provides a mix of detrimental and beneficial microbes, which could be a biologically important source of udder epithelial microbes. Thus, environmental microbiome is not a source of “contaminants” *per se* but rather a source of microbes that adhere to and then inhabit the teat apex and/or canal. Indeed, the environmental microbes within dairy farms have been shown to play an important role in the microbial colonization of the udder (61, 62). Therefore, the use of the word “contamination” to describe the sampling device-associated sequences is not strictly precise, because these sequences could represent microbes that were sourced from the barn air and play an important biological or ecological role within the udder microbiome (61). Further study is needed to discriminate between airborne microbes that contaminate the sample during the actual sampling event and airborne microbes that actually colonize the epithelium of the mammary gland.

### Statistical approaches for identifying the potential source of sequence reads in bovine teat skin and milk microbiome data sets

We applied two-step statistical algorithms for identification of potential contaminant and source sequences in cow teat epithelium and milk samples, using decontam followed by SourceTracker. Decontam identifies contaminants by considering both the frequency of sequence features and the concentration of sample DNA, as well as the prevalence of sequence features in real samples compared with negative controls (43). Hence, decontam requires no knowledge of the source of potential contaminants. Instead, the availability of DNA quantification data and proper preparation of negative controls are needed for accurate prediction by decontam. We demonstrated that decontam was robust in detecting contaminants especially in low-microbial biomass samples, such as cisternal milk and stripped milk, which accounted for 36% and 27%, respectively, of contaminants in the raw sequences. Furthermore, differences in DNA concentration were correlated with differences in the profile of contaminants (Fig. 3), suggesting that the sample microbial biomass is a key factor driving the profile of potential contamination (43). However, contamination is supposed to happen equally in all samples during sample processing, while low microbial biomass samples are at an increased risk of contamination because of a lack of competing biological material. The predominant contaminants that we identified using decontam include many that have been previously reported as contaminants, i.e., *Pseudomonas*, *Streptococcus*, *Clostridium*, and *Turicibacter* (53).

In contrast to decontam, SourceTracker relies on the user to specify both the source and sink samples based on subject-matter knowledge of the study design and sample set, with well-defined contaminating sources providing greater accuracy of prediction than poorly defined source environments (32). Interestingly, the use of SourceTracker after decontam allowed us to identify many more potential contaminants, suggesting that the approaches used by SourceTracker and decontam are sufficiently distinct to detect different types of contaminating and/or source environment microbes. Indeed, the stated goals of the two methods are distinct and our results suggest that using the two tools in sequence is a robust method to profile the sources of contaminating microbes within low-microbial biomass samples such as milk.

It should be noted that SourceTracker assumes that the sink samples are composed wholly of microbes from defined sources. In other words, the sink samples are assumed to be devoid of their own inherent microbiome. As a result, SourceTracker characterizes sequence reads in the sink samples that can’t be associated with a defined source as originating from an “unknown” source. In our analysis, we presumed that these unknown sequences represented the “true” or inherent microbial community within the sink samples (i.e., the milk or skin samples), and therefore, we used only these “unknown” sequences for downstream analysis of the true udder and milk microbiomes. While our extensive use of negative controls was comprehensive in terms of capturing most potential sources of contamination, there could be additional sources that we did not capture in our study design, including host sources such as blood and the gastrointestinal tract. These potential sources of milk-borne bacteria could be particularly important if the cisternal milk microbiome is being colonized by endogenous transfer from the intestine to the microbiota-gut-mammary axis, especially when the integrity of the milk-blood barrier (63) and the intestinal barrier is impaired by inflammation associated with mastitis (6, 64, 65). Furthermore, the negative control samples that we used in this study may not have captured all the bacteria that could have contaminated the milk and skin samples. Additional studies are needed to determine the number and types of negative controls that are needed for sufficient decontamination of bovine milk microbiome data. It is also important to note that neither decontam nor SourceTracker is intended to identify cross-contamination (i.e., sample-to-sample contamination) that may occur during sampling or through well-to-well leakage during library preparation (66). A more extensive study of well placement patterns (including many more blank wells) would be needed to identify these cross-contamination events.

### Dynamics of the udder microbiota ecosystem

Similar to previous studies (67, 68), teat skin microbiome displayed a more diverse and less dispersed structure as compared with milk samples in this study. That might be due to the exposure of teat skin to a relatively stable biotic or abiotic environments, including air, bedding material, milking system, and milker’s contact (4). The teat apex is completely exposed to environmental bacteria, while it serves as an incomplete physical barrier against the entry of environmental microbes into the teat canal (69). Our hypothesized decreasing microbiome gradient from cisternal milk to stripped milk was not supported. We found no significant difference in microbial composition between the teat apex and teat canal samples, supporting the incomplete nature of this physical barrier (70) and indicating that the teat skin microbiome can colonize the teat canal and potentially contaminate the cisternal milk.

Our results suggest that stripped milk is a suitable representative of cisternal milk, with the following caveats: (i) the lack of difference was only observed after applying decontamination procedures; (ii) we only investigated samples from dairy cows without clinical mastitis at the time of sampling; and (iii) all samples were obtained using aseptic sampling techniques (i.e., teat disinfection and prestripping). However, this conclusion may not apply to other contexts (e.g., mastitis positive dairy cows).

### Presence of potential mastitis pathogens with high abundance

After decontamination, we were still able to detect 53 and 68 ASVs representing diverse bacteria in cisternal milk and stripped milk, respectively. However, culture of these milk samples only recovered *Corynebacterium* and *Staphylococcus*, which indicates that the majority of what we detected in the 16S microbiome data was not culturable. This could be because the detected DNA was from non-viable bacteria or either because the bacteria could not grow within our culture conditions or because the bacterial load was below our detection limit of this study (i.e., 100 CFU/mL). The two milk samples that grew *Staphylococcus chromogenes* in our culture conditions were from the same cows that had a high relative abundance of *Staphylococcus* in the sequencing data (Fig. 8), which indicates that the sequence data do correlate with culture results for culturable bacteria.

We detected an average relative abundance of 14.8% for sequences from common potential mastitis pathogens within our skin and milk samples. Bacterial genera from *Staphylococcus*, *Streptococcus*, and *Pseudomonas* were also detected in previous studies on teat skin and cow milk microbiome (4, 15, 71, 72). It is difficult to interpret the relevance of these findings for udder health and dairy cow management because most pathogenic phenotypes are specific to certain species or even strains of bacteria while traditional 16S approaches we and others have used are only able to classify most sequences to the family or genus levels. Shotgun metagenomic sequencing approaches would be a potential alternative for improved resolution of taxa, i.e., to the species or even strain levels, which could also be helpful in identifying potential mastitis pathogens (15, 73). However, species- and strain-level identification from shotgun metagenomic data sets is typically only possible for genomes in relatively high abundance. Given the very high amounts of host DNA in milk, teat canal, and teat apex samples, it would be very expensive to generate enough per-sample shotgun sequence data to recover species- and strain-level details on pathogens within the microbiome. It is also worthy to note that microorganisms grown in the bacterial culture media, e.g., *Staphylococcus chromogenes* and *Corynebacterium* sp., were classified as contaminants (Fig. 3G and 4C) during the decontamination analysis. Previous studies on bacterial culture of milk samples showed that *Staphylococcus* and *Corynebacterium* were contaminants from the teat canal that occurred during the sampling of stripped milk (74, 75). Therefore, the results from bacterial culture of milk samples should be taken cautiously, because contamination could occur during sample collection. Given the importance of these potential mastitis pathogens in the development of intramammary infections and the importance of understanding whether and/or how microbiome results can be associated with udder health, additional more in-depth studies of this nature are warranted.

### Conclusions

After quality control and removal of potential contaminants identified by decontam and SourceTracker, only 20%–24% of the identified sequence reads remained for udder skin and milk microbiome analysis in this study. The source of contamination differed among sample types. Specifically, milk samples were mainly contaminated by DNA from the extraction kit and skin while teat skin microbiomes were mainly contaminated from microorganisms in the air and from the sampling devices. No significant differences in microbial profiles were detected in decontaminated milk samples (cisternal milk vs. stripped milk) or in decontaminated skin samples (teat apex vs. teat canal). However, milk samples displayed a less diverse, more dispersed, and compositionally distinct microbial profile compared with teat skin samples. Across all milk and skin samples, *Actinobacteria* and *Staphylococcus* were the predominant genera, and on average, 14.8% of the sequence reads was classified as being from potential mastitis pathogens. In contract, culture of milk samples only detected the presence of *Staphylococcus* in one cisternal milk sample and *Corynebacterium* and *Staphylococcus* in 50% of stripped milk samples. Our PMA-treated milk samples showed that on average 71.6% of cisternal milk sample reads and 83.6% of stripped milk sample reads were classified as host DNA, which suggests that PMA may be problematic for use in bovine milk molecular diagnostics. Our results highlight the importance of aseptic sampling for udder skin microbiome studies. For future research, we strongly recommend the inclusion of sufficient negative controls to identify potential contamination events and to generate more reliable microbiome data.

## Data Availability

The sequencing data of this article are available at the National Center for Biotechnology Information (NCBI) Sequence Read Archive (SRA) under the BioProject accession number PRJNA983382. The metadata and R scripts necessary to reproduce the analysis presented in this paper are available at https://github.com/TheNoyesLab/Bovine_Milk_Microbiome.

## SUPPLEMENTAL MATERIAL

File S1 msystems.01158-23-s0001.docx Supplemental tables and figures.

File S2 msystems.01158-23-s0002.xlsx All the taxa and the sum of corresponding reads across all samples, for Fig. 3, 4, 7, and 8.

ASM does not own the copyrights to Supplemental Material that may be linked to, or accessed through, an article. The authors have granted ASM a non-exclusive, world-wide license to publish the Supplemental Material files. Please contact the corresponding author directly for reuse.
