# Supplementary material for: The impact of kit, environment, and sampling contamination on the observed microbiome of bovine milk
Source: mSystems. 2024 May 24;9(6):e01158-23. doi: 10.1128/msystems.01158-23 (PMC11237780; doi:10.1128/msystems.01158-23)
Supplement: File S1 — Supplemental tables and figures. [file msystems.01158-23-s0001.docx]

**Supplementary Material**

**The impact of kit, environmental and sampling contamination on the observed microbiome of bovine milk**

C. J. Dean^1†^, Y. Deng^1†^, T. C. Wehri^2^, F. Pena-Mosca^1^, T. Ray^1^, B.A. Crooker^2^, S. M. Godden^1^, L. S. Caixeta^1^^, N.R. Noyes^1*^^

¹Department of Veterinary Population Medicine, University of Minnesota, St. Paul, 55108

²Department of Animal Science, University of Minnesota, St. Paul, 55108

^†^C. J. Dean and Y. Deng contributed equally as co-first authors

^^^L. S. Caixeta and N.R. Noyes contributed equally as co-senior authors

^*^Corresponding author: [nnoyes@umn.edu](mailto:nnoyes@umn.edu)

^*^Noelle R. Noyes, 1988 Fitch Avenue, St. Paul, MN 55108, (612)624-3562

**Table S1.** Characteristics of individual enrolled Holstein dairy cows (n=14)

| Cow ID | Lactation number | DIM | SCC | Milk  (kg/d) | Quarter  sampled | # of cultured distinct organisms (name) | |
| --- | --- | --- | --- | --- | --- | --- | --- |
|  |  |  |  |  |  | Cisternal milk | Stripped milk |
| 2862 | 3 | 306 | 115 | 29 | Right | 0 | 1 (*Corynebacterium*) |
| 2879 | 3 | 368 | 57 | 27 | Right | 0 | 1 (*S. chromogenes*) |
| 2929 | 3 | 100 | 19 | 50 | Left | 0 | >=3 (contaminated) |
| 3012 | 2 | 94 | 22 | 39 | Left | 0 | 0 |
| 3019 | 2 | 105 | 71 | 38 | Right | 1 (*S. chromogenes*) | >=3 (contaminated) |
| 3025 | 2 | 99 | 100 | 43 | Right | 0 | 0 |
| 3033 | 2 | 92 | 18 | 43 | Right | 0 | 1 (*Corynebacterium*) |
| 3037 | 1 | 456 | 44 | 15 | Right | 0 | 0 |
| 3042 | 1 | 349 | 18 | 35 | Left | 0 | 0 |
| 3112 | 1 | 103 | 20 | 35 | Left | 0 | 0 |
| 4776 | 2 | 128 | 57 | 44 | Left | 0 | 2 (*Corynebacterium*; Gram negative organism) |
| 4778 | 2 | 114 | 18 | 42 | Left | 0 | 0 |
| 7485 | 2 | 118 | 174 | 50 | Right | 0 | 0 |
| 7673 | 2 | 122 | 187 | 44 | Right | 0 | 1 (Gram negative rrganism) |

DIM (d), days in milk; SCC (×1,000 cells/mL), somatic cell count was measured in December to screen candidates.

**Table S2**. Theoretical abundance of Zymo standard and actual relative abundance of positive control sample (see Table 1, 16S only, in [ZymoBIOMICS D6310 protocol](https://files.zymoresearch.com/protocols/_d6310_zymobiomics_microbial_community_standard_ii_(log_distribution).pdf) for additional information)

| Taxa | Theoretical  relative abundance | Actual  relative abundance |
| --- | --- | --- |
| *Listeria monocytogenes* | 0.959 | 0.568 |
| *Pseudomonas aeruginosa* | 0.028 | 0.354 |
| *Bacillus subtilis* | 0.012 | 0.040 |
| *Escherichia coli* | 0.00069 | 0.018 |
| *Salmonella enterica* | 0.0007 | 0.014 |
| *Lactobacillus fermentum* | 0.00012 | 0.00308 |
| *Enterococcus faecalis* | 0.0000067 | not detected |
| *Staphylococcus aureus* | 0.0000001 | not detected |
| *Unknown* | not applicable | 0.002 |

**Table S3.** Pairwise PERMANOVA comparisons for the effect of sample type on microbial composition of potential contaminants identified by decontam from teat skin and milk samples (permutations = 999)

|  | R^2^ | F | *P* |
| --- | --- | --- | --- |
| Teat canal_vs_Teat apex | 0.120 | 3.559 | 0.012 |
| Teat canal_vs_Stripped milk | 0.186 | 5.717 | 0.001 |
| Teat canal_vs_Cisternal milk | 0.262 | 9.247 | 0.001 |
| Teat apex_vs_Stripped milk | 0.339 | 12.847 | 0.001 |
| Teat apex_vs_Cisternal milk | 0.407 | 17.876 | 0.001 |
| Stripped milk_vs_Cisternal milk | 0.049 | 1.281 | 0.200 |

**Table S4.** Pairwise PERMANOVA comparison for the effect of sample types on microbial composition of contaminants identified by SourceTracker from teat skin and milk samples (permutations = 999)

|  | R^2^ | F | *P* |
| --- | --- | --- | --- |
| Teat canal_vs_Teat apex | 0.039 | 1.047 | 0.353 |
| Teat canal_vs_Stripped milk | 0.154 | 4.562 | 0.001 |
| Teat canal_vs_Cisternal milk | 0.251 | 8.704 | 0.001 |
| Teat apex_vs_Stripped milk | 0.197 | 6.136 | 0.001 |
| Teat apex_vs_Cisternal milk | 0.298 | 11.030 | 0.001 |
| Stripped milk_vs_Cisternal milk | 0.048 | 1.259 | 0.222 |

**Table S5.** The number of reads (per sample with 95% confidence interval) obtained directly from the sequencer (“raw reads”), and after quality control (QC), after decontam, and after SourceTracker.

|  | **Type** | **mean** | **lower_95% ci** | **upper_95% ci** |
| --- | --- | --- | --- | --- |
| Raw reads | Teat apex | 163,269 | 133,628 | 192,909 |
| Raw reads | Teat canal | 191,423 | 161,850 | 220,996 |
| Raw reads | Stripped milk | 148,636 | 110,995 | 186,277 |
| Raw reads | Cisternal milk | 159,142 | 120,940 | 197,344 |
| After QC | Teat apex | 112,861 | 88,931 | 136,791 |
| After QC | Teat canal | 106,659 | 92,919 | 120,400 |
| After QC | Stripped milk | 93,462 | 74,901 | 112,024 |
| After QC | Cisternal milk | 93,476 | 69,243 | 117,709 |
| After decontam | Teat apex | 108,947 | 85,537 | 132,357 |
| After decontam | Teat canal | 98,873 | 86,602 | 111,144 |
| After decontam | Stripped milk | 53,416 | 30,834 | 75,999 |
| After decontam | Cisternal milk | 36,636 | 23,495 | 49,778 |
| After SourceTracker | Teat apex | 37,753 | 24,379 | 51,127 |
| After SourceTracker | Teat canal | 44,304 | 24,363 | 64,244 |
| After SourceTracker | Stripped milk | 36,346 | 21,393 | 51,299 |
| After SourceTracker | Cisternal milk | 31,726 | 19,575 | 43,877 |

**Table S6.** PERMANOVA comparison and ANOVA analysis of variance for the effect of sample types on microbial composition of animal samples before decontamination, after removing contaminants using decontam and SourceTracker.

|  | PERMANOVA | | |  | ANOVA of Variance | | |
| --- | --- | --- | --- | --- | --- | --- | --- |
|  | R^2^ | F | *P* |  | Mean Sq | F | *P* |
| **Before decontam** | | | | | | | |
| Stripped milk_vs_Cisternal milk | 0.044 | 1.152 | 0.188 |  | 0.019 | 1.593 | 0.219 |
| Teat canal_vs_Teat apex | 0.044 | 1.160 | 0.267 |  | 0.004 | 0.430 | 0.518 |
| **After decontam** | | | | | | | |
| Stripped milk_vs_Cisternal milk | 0.040 | 1.055 | 0.301 |  | 1.68E-05 | 0.042 | 0.840 |
| Teat canal_vs_Teat apex | 0.039 | 1.006 | 0.436 |  | 0.006 | 0.550 | 0.465 |
| **After SourceTracker** | | | | | | | |
| Stripped milk_vs_Cisternal milk | 0.036 | 0.942 | 0.704 |  | 1.04E-05 | 0.100 | 0.755 |
| Teat canal_vs_Teat apex | 0.041 | 1.056 | 0.371 |  | 0.018 | 2.527 | 0.125 |


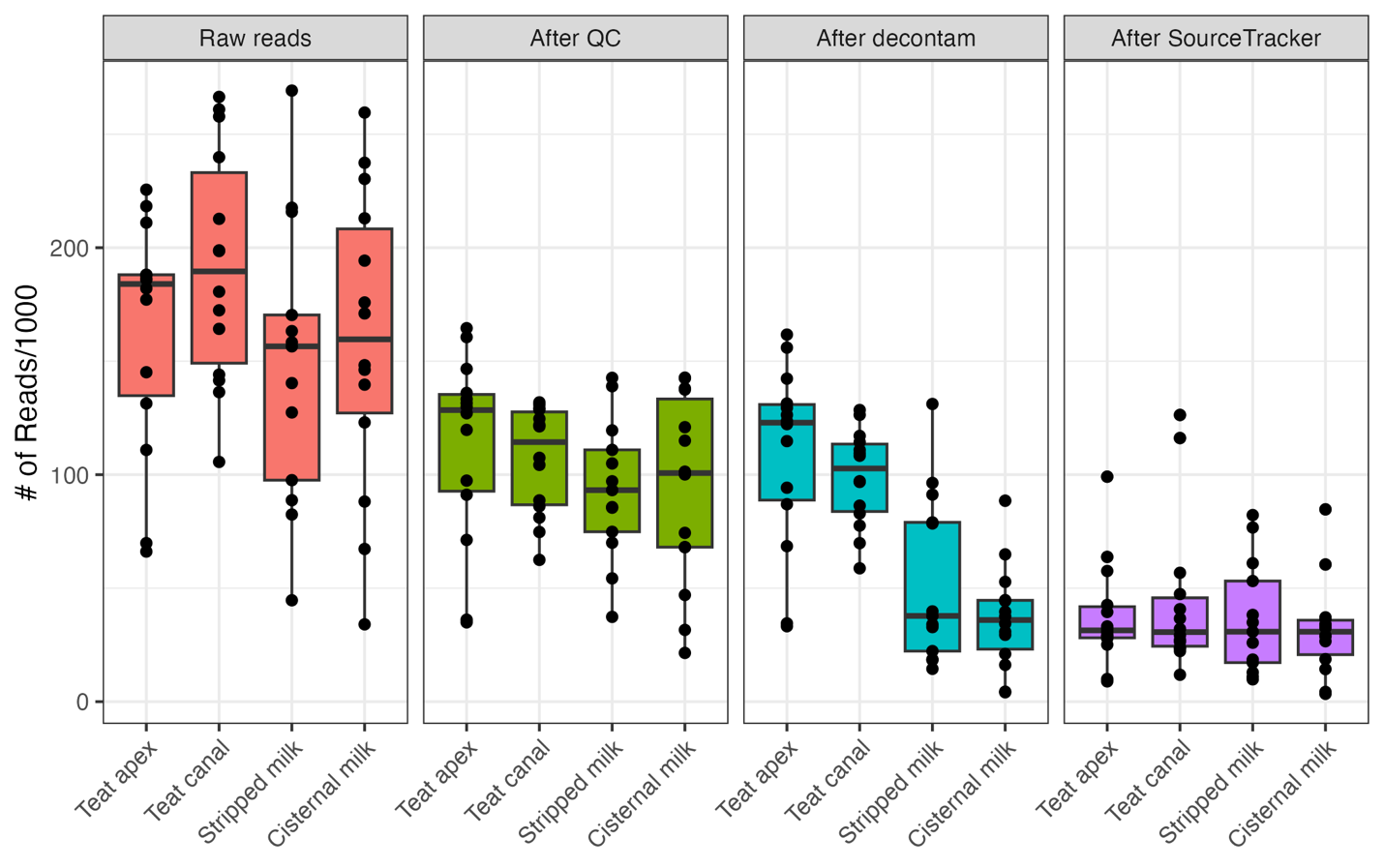


**Figure S1.** The number of reads (per sample) obtained directly from the sequencer (“raw reads”), and after quality control (QC), after decontam, and after SourceTracker, stratified by sample type. Each dot represents a single sample. Box plots represent the median, interquartile range, and outlier values.


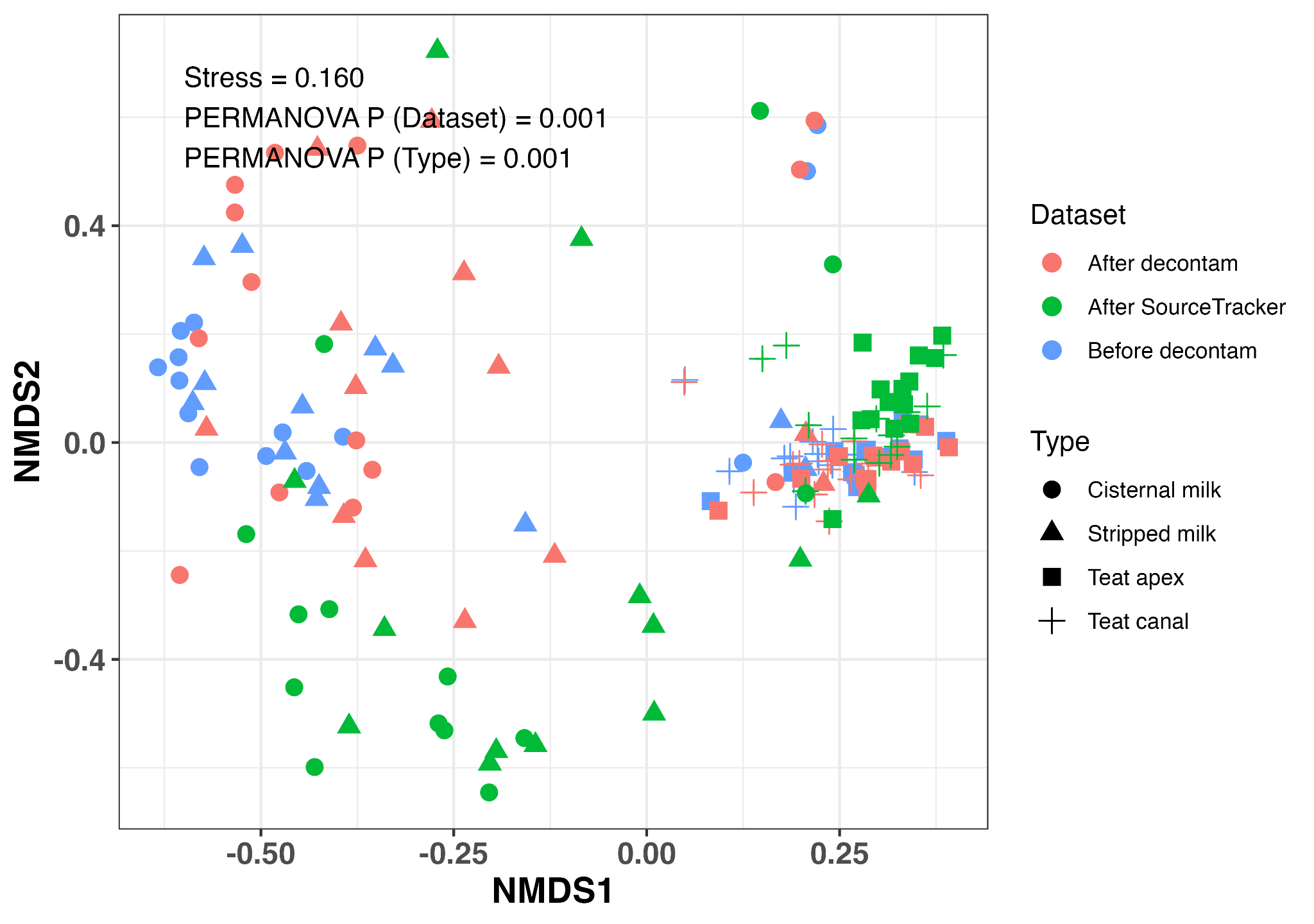


**Figure S2.** NMDS ordination based on Bray-Curtis distance matrix, using the merged dataset of before decontam, after decontam, and after SourceTracker.


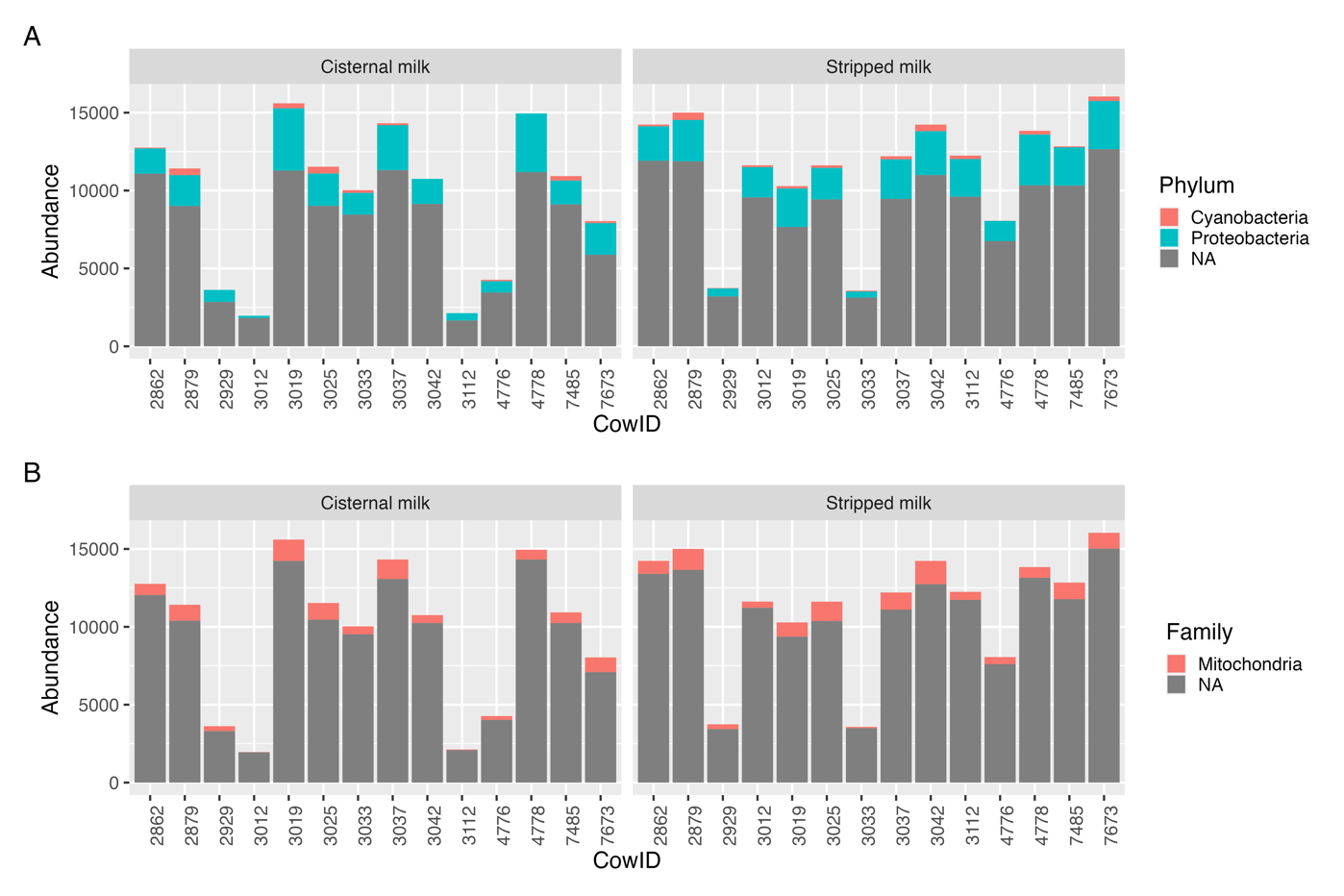


**Figure S3.** The microbial composition of PMA-treated samples on (A) phylum and (B) family taxonomy levels. Sequence features that could not be assigned a taxonomic label at either level were labeled as NA.
